# Targeting the CLK2/SRSF9 splicing axis in prostate cancer leads to decreased ARV7 expression

**DOI:** 10.1002/1878-0261.13728

**Published:** 2024-09-11

**Authors:** Jasper Van Goubergen, Miroslav Peřina, Florian Handle, Elisa Morales, Anika Kremer, Oliver Schmidt, Glen Kristiansen, Marcus V. Cronauer, Frédéric R. Santer

**Affiliations:** ^1^ Division of Experimental Urology, Department of Urology Medical University of Innsbruck Austria; ^2^ Department of Experimental Biology, Faculty of Science Palacký University Olomouc Czech Republic; ^3^ Institute of Pathology, Neuropathology & Molecular Pathology Medical University of Innsbruck Austria; ^4^ Institute of Pathology University Hospital Bonn Germany; ^5^ Institute of Cell Biology, Biocenter Medical University of Innsbruck Austria

**Keywords:** 3′ untranslated region, allele‐specific regulation, androgen receptor splice variant 7, dual specificity protein kinase CLK2, serine/arginine‐family of splicing factors, splicing inhibitors

## Abstract

In advanced prostate cancer (PC), in particular after acquisition of resistance to androgen receptor (AR) signaling inhibitors (ARSI), upregulation of AR splice variants compromises endocrine therapy efficiency. Androgen receptor splice variant‐7 (ARV7) is clinically the most relevant and has a distinct 3′ untranslated region (3′UTR) compared to the AR full‐length variant, suggesting a unique post‐transcriptional regulation. Here, we set out to evaluate the applicability of the *ARV7* 3′UTR as a therapy target. A common single nucleotide polymorphism, rs5918762, was found to affect the splicing rate and thus the expression of *ARV7* in cellular models and patient specimens. Serine/arginine‐rich splicing factor 9 (SRSF9) was found to bind to and increase the inclusion of the cryptic exon 3 of *ARV7* during the splicing process in the alternative C allele of rs5918762. The dual specificity protein kinase CLK2 interferes with the activity of SRSF9 by regulating its expression. Inhibition of the Cdc2‐like kinase (CLK) family by the small molecules cirtuvivint or lorecivivint results in the decreased expression of *ARV7*. Both inhibitors show potent anti‐proliferative effects in enzalutamide‐treated or ‐naive PC models. Thus, targeting aberrant alternative splicing at the 3′UTR of *ARV7* by disturbing the CLK2/SRSF9 axis might be a valuable therapeutic approach in late stage, ARSI‐resistant PC.

## Graphical abstract

In advanced prostate cancer (PC), resistance to AR signaling inhibitors upregulates splice variant ARV7. SNP rs5918762 located in the 3′ untranslated region of cryptic exon 3 affects ARV7 expression by involving the splice factor SRSF9. CLK2 regulates SRSF9, and the pharmacological inhibition of CLK2 reduces ARV7 levels. Thus, targeting alternative splicing in advanced PC may improve current treatment strategies.

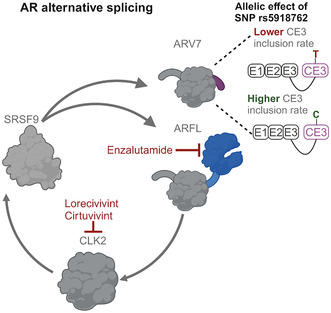

**Abbreviations**
3′ UTR: 3′ untranslated region
AFR: African
AR: androgen receptor
ARFL: androgen receptor full length
ARSI: androgen receptor signaling inhibitors
ARV7: androgen receptor variant 7
ASN: Asian
CDS: coding sequence
CE3: cryptic exon 3
Cir: cirtuvivint
CLK: Cdc2‐like kinase
CRPC: castration‐resistant prostate cancer
E: exon
Enza: enzalutamide
EnzaR: enzalutamide resistant
gRNA: guide RNA
GSEA: gene set enrichment analysis
hnRNP: heterogeneous nuclear ribonucleoprotein
HSA: highest single agent
Lor: lorecivivint
m6A: N6‐methyladenosine
MAF: minor allele frequency
n.t.: non targeting
NHEJ: non‐homologous end joining
PC: prostate cancer
qPCR: quantitative polymerase chain reaction
RBP: RNA‐binding protein
SEM: standard error of means
SNP: single nucleotide polymorphism
snRNP: small nuclear ribonuclear particles
SRPM: splice reads per million
SRSF: serine/arginine‐rich splicing factor
STR: short tandem repeat
TIDE: tracking of indels by decomposition
TURP: trans‐urethral resection of the prostate
YB1: Y‐box protein 1

## 1 Introduction

Castration‐resistant prostate cancer (CRPC) is the advanced stage of prostate cancer (PC) and arises after failure of endocrine therapy. Despite therapeutic approaches with second‐generation androgen receptor signaling inhibitors (ARSI), e.g., enzalutamide (Enza) or abiraterone acetate, this stage remains lethal as resistance to these drugs invariably occur. One of the underlying molecular adaptations of the cancer cell is the expression of AR variants that, devoid of their ligand‐binding domain, are transcriptionally active in an androgen and anti‐androgen independent manner and may drive therapy resistance [1]. Clinically the most frequent and thus relevant variant is ARV7 (also termed AR3) as its presence in the nucleus of circulating tumor cells is used to stratify CRPC patients for taxane‐based chemotherapy (ARV7‐positive) or treatment with ARSI (ARV7‐negative) [2]. ARV7 arises by alternative splicing from the same pre‐mRNA as the full length AR (ARFL) variant. While ARFL is encoded by exons (E) 1–8, ARV7 is formed from E1–3 and by addition of cryptic E3 (CE3) localized in the intronic region between E3 and E4 [1]. As a result, ARFL and ARV7 transcripts possess distinct 3′ untranslated regions (3′UTRs) encoded by E8 and CE3, respectively. In general, 3′UTRs are important regions for post‐transcriptional regulation including mRNA stability, localization, and translation [3]. Single nucleotide polymorphisms (SNPs) located in 3′UTRs have been shown to influence the expression of genes [4] and, together with clinical factors, to impact survival prediction and treatment response [5].

The spliceosome is one of the most complex, dynamic molecular machineries in our cells involving small nuclear ribonucleoprotein particles (snRNPs) and numerous auxiliary proteins. Splice factors are RNA‐binding proteins (RBPs) that can bind to intronic or exonic splicing enhancers or silencers. In cancer cells, aberrant alternative splicing is a common process that was documented for every hallmark of cancer [6]. Its underlying mechanism is the altered expression and activation of splicing factors such as members of the heterogeneous nuclear ribonucleoprotein (hnRNP) proteins and the SR (serine/arginine‐rich) family consisting of SRSF1‐12 and TRA2A/B. To date, from the SR family only SRSF1 [7] and SRSF3 [8] have been associated with ARV7 splicing, while the role of other family members in this process is unknown. However, the increased expression of SRSF2, ‐3, ‐4, ‐5, ‐9, and ‐10 has been found in PC tumor vs. nontumor adjacent specimens [8] showing that PC is associated with changes in the splicing process. Targeting alternative splicing to decrease ARV7 expression has been proposed. However, the understanding of the splicing process in general, and of the *AR* gene in particular, is currently too little understood to develop effective splicing inhibitors counteracting its aberrant alternative splicing.

Here, we set out to better characterize the regulatory functions of ARV7's 3′UTR. To ensure clinical relevance, we selected a common SNP, rs5918762, to investigate the allele‐specific binding of RBPs. SRSF9 was found to bind to the proximal 3′UTR of ARV7 and to mediate its allele‐specific splicing. The expression of this RBP was regulated by the dual specificity protein kinase CLK2. Finally, small molecule inhibitors directed against CLK2 were characterized for their efficiency in decreasing ARV7 expression and as a possible combination therapy added to Enza treatment.

## 2 Materials and methods

### 2.1 Cell culture and transfection

Cell lines LNCaP‐FGC (RRID: CVCL_1379), 22Rv1 (RRID: CVCL_1045), PC‐3 (RRID: CRL‐1435) and MDA PCa 2b (RRID: CVCL_4748) were purchased and cultured as recommended by ATCC. HEK293FT (RRID: CVCL_6911) were purchased and cultured as recommended by ThermoFisher Scientific. The DuCaP cell line, which was a kind gift from J. Schalken (Radboud UMC, Nijmegen, the Netherlands), and PC‐3 were cultured in RPMI 1640, supplemented with 10% (v/v) fetal bovine serum (FBS), 1% (v/v) Pen/Strep (10 000 units·mL^−1^ Penicillin; 10 mg·mL^−1^ Streptomycin) and 1% (v/v) GlutaMax. 22Rv1 and LNCaP were cultivated in the same medium with supplements plus additionally 1% (v/v) 1 m HEPES, 1.4% (v/v) 250 g·L^−1^ d‐glucose and 1% (v/v) 100 mm Na‐pyruvate were added. MDA PCa 2b were cultivated in an F‐12K medium, supplemented with 20% (v/v) FBS, 1% (v/v) Pen/Strep (10 000 Units·mL^−1^ penicillin; 10 mg·mL^−1^ streptomycin), 1% (v/v) GlutaMax, 25 ng·mL^−1^ cholera toxin, 10 ng·mL^−1^ EGF, 5 μm phosphoethanolamine, 100 pg·mL^−1^ hydroxycortisone, 45 nm selenious acid, and 5 μg·mL^−1^ insulin. Cell line authentication was performed on all cell lines by STR analysis; since DuCaP originates from the same patient, the STR profiles for VCaP were used. All experiments were performed with mycoplasma‐free cell lines, which was controlled by routine testing for mycoplasma contaminations by isothermal PCR. Stable transfections (i.e., 22Rv1‐Cas9, DuCaP‐Cas9, LNCaP‐CLK2, LNCaP‐SRSF9, 22Rv1‐Flag‐ARV7‐3′UTR) were done by lentiviral transductions of lentiCRISPR v2 (a gift from F. Zhang (Addgene plasmid # 52961; http://n2t.net/addgene:52961; RRID: Addgene_52961) [9]), pLenti6‐CLK2, pLenti6‐SRSF9, and pLenti6‐Flag‐ARV7‐3′UTR respectively, and subsequent selection with puromycin or blasticidin, respectively. Transient transfections were done with 40 pmol of sgRNAs with 7.5 μL Lipofectamine RNAiMAX per 6‐well. Plasmid transfections were performed with 2 μg plasmid DNA in a 1 : 3 ratio with X‐tremeGENE HP DNA Transfection Reagent. Cells were harvested after 72 h post‐transfection unless indicated otherwise.

### 2.2 Drug response

Dose‐dependent drug responses for Cirtuvivint (Cir) and Lorecivivint (Lor) were monitored by Incuycte confluence measurements. Briefly, cells were seeded in 96‐well plates and allowed to attach overnight. On the following day, cells were treated with increasing concentrations [ranging from 0 (vehicle) to 10 μm] of the drug. IC50 determination was assessed 72 h post‐treatment. For Lor/Enza combination treatment, drug concentrations ranged from 0 (vehicle) to 2 μm for Lor and 0 (vehicle) to 200 μm for Enza.

### 2.3 RNA isolation, reverse transcription and qPCR

Total RNA was isolated using the BLIRT ExtractMe Total RNA kit and reverse transcribed into cDNA with the LunaScript RT SuperMix according to manufacturers' recommendations. cDNA samples were diluted 1 : 10 with nuclease‐free water and 1 μL of cDNA was measured with the Luna Universal Probe qPCR Master Mix with 400 nm of forward and reverse primer and 200 nm Taq‐man probe, all specific for the gene of interest (GOI). Thermo‐cycling conditions were as recommended by the manufacturer (CFX Connect; Bio‐Rad, Vienna, Austria) and *C*_t_ values were assessed using regression analysis mode. All data were normalized to the geometric mean of three reference genes (HPRT1, TBP, and HMBS). Relative expression was calculated according to the ΔΔ*C*_t_ method. Results are shown as relative bar plots of the respective GOI normalized to the geometric mean of three housekeeping genes.

### 2.4 Immunoblotting and phosphate‐affinity (Phos‐tag) gel electrophoresis

Immunoblotting was previously described [10]. Band detection was done with fluorescence‐labeled, secondary antibodies on a Li‐Cor Odyssey CLx scanner (Bad Homburg, Germany), or for more sensitive detection with HRP‐labeled, secondary antibodies using Femto Maximum Sensitivity ECL substrate on a Bio‐Rad ChemiDoc MP imaging system. For Phosphate‐affinity gel electrophoresis, Laemmli buffer supplemented with 1% β‐mercaptoethanol was used to prepare samples [11], which were quantity adjusted based on GAPDH levels of a normal immunoblot. Fifty micromolar Phos‐tag and 100 μm MnCl_2_ supplemented to 12.5% polyacrylamide gels were used for the separation of phosphorylated SRSF9. After electrophoresis, gels were rinsed in transfer buffer containing 10 mm EDTA for 20 min, followed by wet electroblotting to nitrocellulose membranes. Controls for unphosphorylated protein were generated by incubating 40 μg of lysate with > 400 U of lambda protein phosphatase (λ‐PPase) for 1 h at 30 °C. Uncropped immunoblots of all experiments performed within this study can be found in Fig. S1.

### 2.5 Genome editing and editing efficiency assessment

To generate indels in the 3′UTR of ARV7, 22Rv1‐cas9 and DuCaP‐cas9 were transfected with different sgRNAs. The editing efficiency was determined on cDNA 72 h post‐transfection by TIDE analysis [12]. Briefly, cDNA samples were amplified by PCR, purified and Sanger sequenced by both forward and reverse primer. ABI traces were then uploaded to the TIDE portal along with sequences from the nontargeting (n.t.) sgRNA‐transfected sample used for comparison by the software.

### 2.6 CLIP‐qPCR

Ten million cells were seeded in a 150 mm culture plate. The following day, cells were UV crosslinked by 400 mJ·cm^−2^ in a Stratagene UV Stratalinker 1800. RIPA buffer [1% (v/v) Triton X‐100, 0.5% (w/v) sodium deoxycholate, 150 mm sodium chloride, 50 mm Tris, pH 8.0] supplemented with recombinant RNase inhibitor, was used to lyse the cells. Lysate was then subjected to pre‐clearance by the addition of magnetic protein G beads (1 h, 4 °C). Cleared lysates were incubated with 4 μg anti‐SRSF9 per 500 μg lysate or with the corresponding amount of mouse IgG2α isotype control (overnight, 4 °C), followed by addition of fresh magnetic protein G beads. After incubation, beads were washed twice with wash buffer I [(20 mm Tris/HCl pH 8, 150 mm NaCl, 2 mm EDTA, 0.1% (w/v) SDS, 1% (v/v) Triton‐X‐100, 1 : 200 (v/v) protease inhibitor cocktail and recombinant RNase inhibitor (1 U·μL^−1^)] and twice with wash buffer II (same composition as wash buffer I, but concentration of NaCl was increased to 500 mm). Albeit these washing conditions removed most of the unspecific binding of SRSF9 to IgG‐coupled beads (likely a result of SRSF9 binding directly to the beads), it also impacted the binding of SRSF9 to its antibody and thus SRSF9 pull‐down efficiency. For protein analysis purposes, elution was performed by boiling samples in an LDS sample buffer. For RNA analysis purposes, elution was performed by subjecting samples to PCR grade proteinase K digestion (15 min, 55 °C). RNase T1 partial digestion step was performed before the formation of the immunocomplexes at a concentration of 1 U·μL^−1^ for 5 min at 22 °C. RNA was then purified using RNA Clean & Concentrator‐5 kit (Zymo Research, Distributor: Szabo‐Scandic, Vienna, Austria) and *ARFL*/*V7* binding was assessed by qPCR using TaqMan Probes. Detailed mapping of SRSF9 binding on the ARV7 3′UTR was performed by qPCR primer walking.

### 2.7 Expression quantitative trait loci study and the gene expression in patient samples

A previously reported androgen deprivation therapy PC cohort consisting of specimens (*n* = 51) from trans‐urethral resections of the prostate (TURP) [13] was genotyped for rs5918762 by Sanger sequencing. To this end, deparaffinization of the formalin‐fixed, paraffin‐embedded samples was done by Xylene at 56 °C for 2 min, followed by two ethanol washes. Quick Extract FFPE DNA Extraction Solution was added to the sample and gDNA was PCR amplified using eQTL.FOR/REV primers. Amplicons were purified, Sanger sequenced and ABI traces were aligned in Snapgene to call the allelic status. For gene expression studies, previously extracted total RNA/cDNA samples were used [13]. The expressions of *ARV7*, *ARFL*, *CLK2*, and *SRSF9* were measured by SybrGreen qPCR on a 7500 Fast Real‐Time PCR System (Applied Biosystems, Thermo Fisher Scientific, Vienna, Austria). Details of the patient cohort can be found in Table S1.

### 2.8 Roadblock decay assay

22Rv1 were stably transduced by lentiviral particles containing pLenti6‐Flag‐ARV7‐3′UTR‐rs5918762 T or C, respectively. Roadblock mRNA decay assay was performed as previously reported [14]. Briefly, attached cells were incubated with 400 μm 4‐Thiouridin (4sU) for 0–8 h and harvested by addition of the first cell lysing buffer of BLIRT ExtractMe Total RNA kit (LabConsulting, Vienna, Austria). One microgram of isolated RNA was modified by adding 3 μL/20 μL *N*‐ethylmaleimid (NEM) at 42 °C for 90 min adding bulky adducts to newly synthesized mRNA moieties with 4sU incorporation. For normalization, one aliquot of each sample was incubated without NEM. After purification with the RNA Clean & Concentrator‐5 kit, mRNA was reverse transcribed using the LunaScript RT Master Mix Primer free kit with d(T)23VN primers. qPCR directed against the Flag‐taq was performed and each sample was normalized to its respective NEM‐untreated control.

### 2.9 Minigene reporter assay

The assay was updated from a previously reported assay [15]. Instead of using reverse transcriptase PCR, qPCR was used. For normalization between the samples, pcDNA3.1‐GFP [a gift from B. Huang (Addgene plasmid #70219; http://n2t.net/addgene:70219; RRID: Addgene_70219)] [16] was co‐transfected at a ratio of 3 : 1 and measured by qPCR using primer/probes directed against GFP.

### 2.10 Construction of plasmids

The ARV7‐minigene assay was constructed as follows. Fibroblast gDNA was used as a template to amplify E3, CE3, E4, and their respective flanking 500 intronic nucleotides. Fragments were fused together by overlap‐extension‐PCR using Phusion High‐Fidelity DNA Polymerase. This single fragment was then cloned into pcDNA3.1 by in‐fusion cloning using In‐Fusion^®^ Snap Assembly Master Mix. The rs5918762 alternative C allele was introduced using the QuikChange Lightning Site‐Directed Mutagenesis kit (Agilent, Santa Clara, CA, USA).

pmiR‐GLO‐ARV7 3′UTR was also constructed by in‐fusion cloning. In short, the ARV7 minigene assay was the starting material to PCR amplify ARV7's 3′UTR from CE3. This amplicon was then inserted by in‐fusion cloning in pmirGLO. Alternative alleles of all studied polymorphisms were introduced using the QuikChange Lightning Site‐Directed Mutagenesis kit.

To generate pLenti6‐CLK2, PCR was done on pDONR223‐CLK2 [a gift from W. Hahn and D. Root (Addgene plasmid # 23685; http://n2t.net/addgene:23685; RRID: Addgene_23685)] [17] to attach attB sites to the CLK2 coding sequence. Gateway BP reaction was performed with pDONR221 followed by LR reaction with pLENTI6/V5‐DEST. The C‐terminal V5‐taq was after the Stop codon of CLK2.

pLenti‐SRSF9 was cloned by the same principle as pLenti6‐CLK2 using pSRSF9‐mCherry as a starting material. This plasmid was a kind gift of A. Girstun, University of Warsaw, Poland [18]. SRSF9's coding sequence was amplified with primers having attB overhangs, followed by Gateway BP and LR reactions.

pLenti6‐Flag‐ARV7‐3′UTR was constructed from pEGFP‐ARV7 (a kind gift of J. Céraline, University of Strasbourg, France) and pcDNA3.1 ARV7 minigene plasmids. Firstly, EGFP was replaced to generate pFlag‐ARV7. Then, the Flag‐ARV7 CDS from pFlag‐ARV7 and the 3′UTR from pcDNA3.1 ARV7 minigene were PCR amplified and inserted in pENTR221 by in‐fusion cloning, which generated pENTR221‐Flag‐ARV7‐3′UTR. The allelic status of rs5918762 was then changed by site‐directed mutagenesis. Finally, both allele‐differing pENTR221‐Flag‐ARV7‐3′UTR were shuttled by Gateway LR recombination to generate pLenti6‐Flag‐ARV7‐3′UTR‐rs5918762 C or T.

### 2.11 Sanger sequencing

Sanger sequencing was done by Microsynth AG (Balgach, Switzerland). All plasmids were sequenced to confirm the accuracy of the constructs. For aligning the sequences of isolated bands (Fig. S2G), overlapping ABI traces were decomposed using the Mixed Sequence Reader software [19].

### 2.12 Bioinformatical analyses

Kaplan–Meier plots were generated using data from The Cancer Genome Atlas (TCGA) [20] in GEPIA [21]. Differential gene expression analyses for expression cohorts of AR‐V7 and SRSF9 and copy number alteration analysis were downloaded from the SU2C dataset through cBioportal.org. Short‐read bulk RNA‐seq analyses for LNCaP and MDA PCa 2b enzalutamide resistant cells and for Lorecivivint‐treated 22Rv1 were performed by Novogene using their strand‐specific poly‐A enrichment pipeline and sequenced on a Novaseq 6000 machine (Illumina, San Diego, CA, USA) using the 150 paired end mode. Bioinformatics analysis was performed in r using the rsubread package for alignment/feature counting (Gencode genome release 43) and the edger package for differential gene expression analysis. Pathway analysis was performed with the camera function of edger. Long‐read bulk RNA‐sequencing analysis was performed by CD Genomics using the SQK‐PCS109 cDNA sequencing kit on a PromethION machine using the guppy (v6.4.6) (Oxford Nanopore Technologies, Oxford, UK) basecaller. Bioinformatic analysis was performed with the epi2me software (v5.1.8) (Oxford Nanopore Technologies) using the wf‐transcriptomes pipeline.

### 2.13 Statistical analyses

All experiments were performed in triplicates at the least. Graphs and statistical analysis were performed using graphpad prism v9 (GraphPad Software, Boston, MA, USA) and rstudio (version: 1.4.1106) (Posit PBC, Boston, MA, USA). Depending on experimental design Student's *t*‐test, one‐way ANOVA, or Wilcoxon–Mann–Whitney test were performed. Results are always displayed as mean ± standard error of means (SEM). qPCR, immunoblot densitometry and IC50 calculation results were intra‐replicate‐normalized by dividing each measurement by the mean of that replicate. Statistical significant *P*‐values are encoded as follows: *P < 0.05, **P < 0.01, ***P < 0.001, ****P < 0.0001.

### 2.14 Study approval

The analysis of patient samples was approved by the ethical committee of the University of Bonn (Nr. 124/19) and was a retrospective study with tissue samples from anonymized patients collected from 2003 to 2018 at the Institute of Pathology of the University of Bonn and who are not identifiable from the data set. Written informed consent is not available for the patients of this data set. The study was conducted in accordance with the Declaration of Helsinki.

## 3 Results

### 3.1 Genomic variations in the 3′UTR decrease the expression of ARV7

**Fig. 1:**
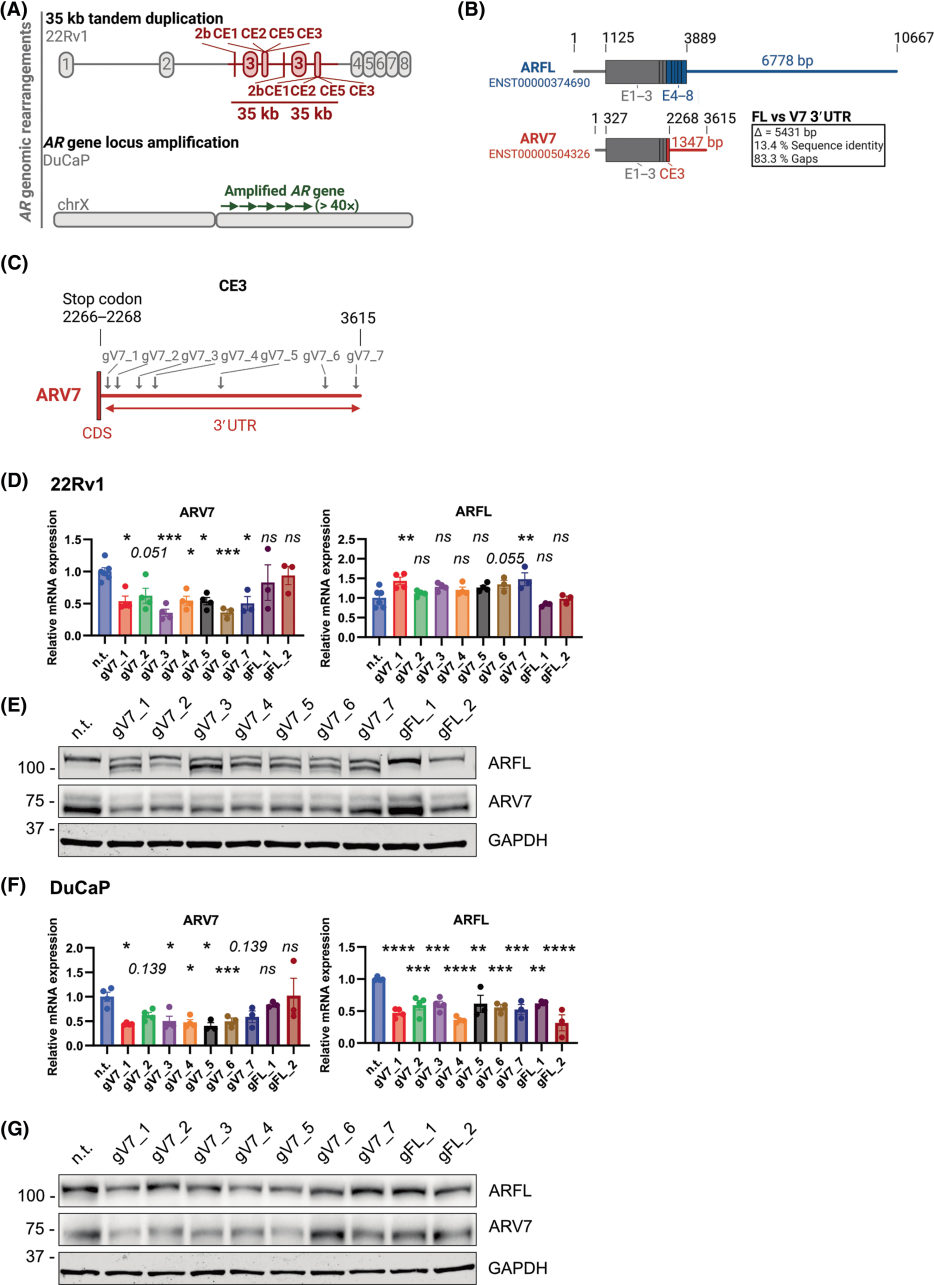
An intact 3′UTR sequence is important for the ARV7 mRNA expression. (A) Overview of the genomic rearrangements in the ARV7‐positive cell lines 22Rv1 and DuCaP. 22Rv1 harbors a tandem duplication of E3 in 22Rv1 and a copy number amplification of the AR gene locus in DuCaP was assessed in Fig. S2A. CE, cryptic exon; ChrX, chromosome X; E, exon. (B) Overview of ARFL and ARV7 transcripts as annotated in Ensembl genome browser 112. Scheme shows the alignment of both transcripts and a sequence identity comparison of the respective 3′ untranslated regions (3′UTRs). FL, full length; V7, variant 7. (C) Scheme depicting the positions of guide RNAs (gRNAs) gV7_1 – gV7_7 targeting the 3′UTR of ARV7. Positions of gFL_1 and gFL_2 can be found in Fig. S2B. CDS, coding sequence. (D–G) 22Rv1‐cas9 and DuCaP‐cas9 were transfected with gRNAs targeting the 3′UTRs of ARV7 and ARFL, or nontargeting (n.t.) gRNA. Three days post‐transfection cells were analyzed for ARV7 and ARFL expression by qPCR (D, 22Rv1; F, DuCaP) and immunoblotting (E, 22Rv1; G, DuCaP). Statistical testing was done by one‐way ANOVA (mean ± SEM; *n* values for panel D: n.t. = 5, gV7_1–5 = 4, gV7_6–7 and gFL1_2 = 3; for panel E: n.t. = 4, gV7_1–7 and gFL_1–2 = 3, for panel F: n.t. and gV7_1–4 = 4, gV7_5–7 and gFL1_2 = 3, for panel G: all = 4; ns = *P* > 0.05, *P < 0.05, **P < 0.01, ***P < 0.001, ****P < 0.0001). (E, G) A representative blot is shown. Dot plots of relative protein expressions can be found in Fig. S2C,E. (D–G) Editing efficiencies of gRNAs as assessed by the TIDE analysis can be found in Fig. S2D,F.

High expression of ARV7 in PC specimens is usually coupled to copy‐neutral or amplified structural rearrangements of the *AR* gene [22, 23]. For our study, we selected as a model for copy‐neutral rearrangement the cell line 22Rv1, which has previously been characterized to contain a tandem duplication of a ~ 35 kb genomic region carrying exons 2b, 3, and CE1, ‐2, ‐5, and ‐3 resulting in ARFL transcripts with two consecutive copies of E3 [24] (Fig. 1A). In the DuCaP cell line we detected ~ 49 *AR* gene locus copies by qPCR to genomic DNA (Fig. 1A, Fig. S2A), thus serving as a model for amplified rearrangement. While the 3′UTR of ARFL is encoded by E8 of the *AR* gene, ARV7 possesses a distinct 3′UTR encoded by CE3. Sequence identity comparisons of both 3′UTRs showed only marginal similarity (Fig. 1B) suggesting that both transcripts might be regulated through different post‐transcriptional mechanisms acting on the respective 3′UTRs. Furthermore, this fact might be exploited therapeutically to target ARV7 specifically. Thus, we set out to elucidate the importance of the 3′UTR sequence for post‐transcriptional regulation of *ARV7* transcripts. Using CRISPR/Cas9‐mediated indel generation by nonhomologous end joining (NHEJ) repair, we introduced genomic variations in the proximal, middle, and distal 3′UTR of *ARV7* in both cell lines (Fig. 1C). For controls, we used a no.t. gRNA, as well as two gRNAs targeting *ARFL*'s 3′UTR (Fig. S2B). In 22Rv1, this approach led to a reduction of *ARV7* mRNA (Fig. 1D) and protein (Fig. 1E, Fig. S2C) when the 3′UTR of *ARV7* was targeted. The decreased expression of ARV7 was comparable with or better than the estimated gRNA targeting efficiency evaluated by TIDE analysis on corresponding cDNA samples, except for gV7_4 (Fig. S2D). *ARFL* mRNA levels showed slightly increased expression (Fig. 1D), while in immunoblotting analysis the anti‐ARFL antibody detected a second, faster migrating band upon targeting *ARV7*'s 3′UTR (Fig. 1E, Fig. S2C). Decreased *ARV7* mRNA (Fig. 1F) and protein (Fig. 1G, Fig. S2E) expression levels were also detected in DuCaP cells upon targeting ARV7's 3′UTR, and the amplitude of decrease was higher than the gRNA targeting efficiency (Fig. S2F). Interestingly, *ARFL* mRNA and protein expression levels were also decreased in the DuCaP cell line, which was also observed in the case of gRNAs targeting *ARFL*'s 3′UTR. To analyze the underlying molecular mechanisms for these discordant results we performed PCR analyses on cDNA in 22Rv1 and gDNA in DuCaP after transfection with n.t. and gV7_4 gRNAs (Fig. S2A,G). In 22Rv1, transfection of gV7_4 gRNA led to a partial de‐duplication of the amplified ~ 35 kb locus, which likely corresponds to the slower migrating ARFL band in immunoblots (Fig. S2G, Fig. 1E). However, as transcription of ARV7 is in most cases terminated at the first polyA signal of the two copies of CE3, this de‐duplication does not affect expression of *ARV7* mRNA (Fig. S2G). Therefore, we concluded that the observed downregulation of *ARV7* mRNA and protein upon targeting its 3′UTR in 22Rv1 is the result of a disturbed post‐transcriptional mechanism. In DuCaP, targeting of *ARV7*'s 3′UTR by gV7_4 resulted in ~ 50% decrease in *AR* copy numbers (Fig. S2A) explaining the observed decreased expression of ARFL (Fig. 1F,G, Fig. S2E). Whether the observed decrease of ARV7 expression in DuCaP is a result of decreased *AR* copy numbers, or of a disturbed post‐transcriptional mechanism, or a combination of both remains unclear. In summary, we concluded that, at least in 22Rv1, an intact *ARV7* 3′UTR sequence is important for the expression of ARV7 and that various post‐transcriptional mechanisms may act on the proximal and distal 3′UTR of *ARV7* regulating its expression.

### 3.2 SNP rs5918762 regulates the expression of ARV7 through a splicing mechanism

**Fig. 2:**
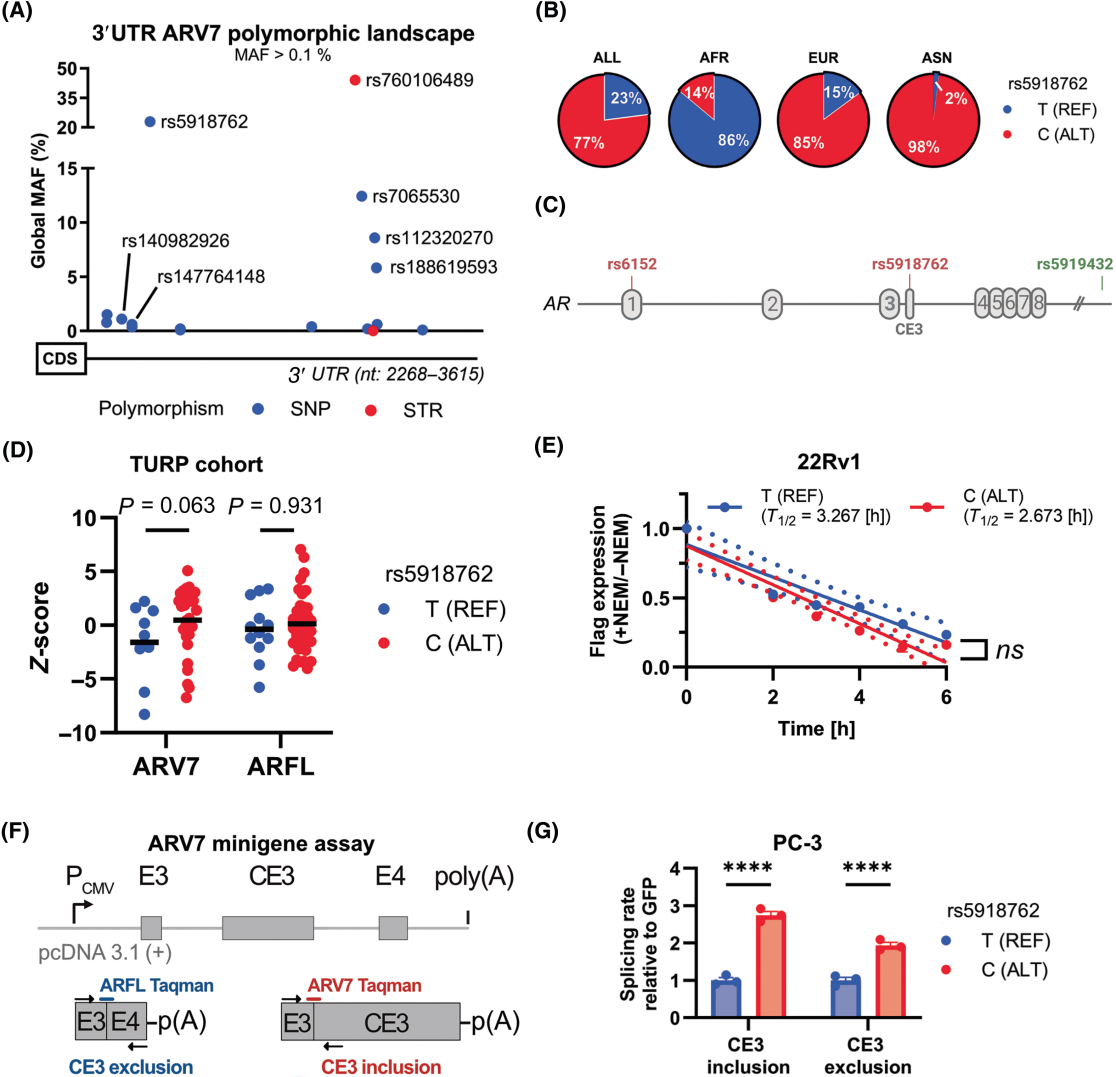
rs5918762 is a common SNP located in the 3′UTR of ARV7 having a role in AR alternative splicing. (A) Scheme depicting the polymorphic landscape of ARV7's 3′ untranslated region (3′UTR) showing variations with a minor allele frequency (MAF) > 0.1%. Data was obtained from Ensembl genome browser 112. CDS, coding sequence; SNP, single nucleotide polymorphism; STR, short tandem repeat. (B) Pie charts depicting the aggregated allelic frequencies (ALFA dataset) of rs5918762 as retrieved from dbGAP for selected populations: AFR, African; ALL, global; ALT, alternative; ASN, Asian; EUR, European; REF, reference. (C) Scheme depicting the location of SNPs located in exonic regions of the haplotype identified by taq SNP rs5919432. CE3, cryptic exon 3. (D) Expression quantitative trait loci (eQTL) analysis of rs5918762 in a trans‐urethral resection of the prostate (TURP) cohort (*n* = 51) of PC patients with previous endocrine therapy. ARV7 and ARFL mRNA expression levels were assessed by qPCR as described in Section 2. Genotyping was done by Sanger sequencing on corresponding tissue sections. Expression levels are shown as *Z*‐scores and statistical testing was done by Wilcoxon‐Mann–Whitney test. (E) mRNA decay assay for ARV7 comparing the different allelic states of rs5918762. Flag‐ARV7‐3′UTR‐rs5918762 C/T constructs were stably transfected into 22Rv1 and half life of ARV7 mRNA was measured by the ROADBLOCK method and qPCR against Flag‐tag. Samples were normalized to *N*‐ethylmaleimide (NEM)‐untreated samples. Dashed lines indicate 95% confidence intervals. Student's *t*‐test was used to compare half lives (mean ± 95% confidence interval, *n* = 3). (F) Scheme depicting the ARV7 minigene assay used in this study. Exons are flanked by their respective 500 up‐ or downstream intronic sequences. In qPCR analysis specific reverse primers and Taqman probes were used to discriminate between CE3 exclusion and inclusion, while the same forward primer was used. (G) ARV7 minigene assay in PC‐3 cells comparing the impact of the two different allelic states of rs5918762. Results were normalized by co‐transfection of GFP and analyzed statistically by Student's *t*‐test (mean ± SEM, *n* = 3, ****P < 0.0001).

Since an intact 3′UTR sequence is crucial for the expression of ARV7, we were interested whether genetic polymorphisms may also affect its post‐transcriptional regulation. The 3′UTR of ARV7 contains several SNPs and short tandem repeats (STRs) with a minor allele frequency (MAF) > 0.1% (Fig. 2A). Of potential clinical interest due to their high MAF are in the proximal 3′UTR the SNP rs5918762, and in the distal region the STR rs760106489 and the SNPs rs7065530, rs112320270, and rs188619593. For the proximal rs5918762, population‐associated changes in MAF are documented for the African (AFR) population possessing 86% the reference T allele, while in contrast in Asians (ASN) the alternative C allele is dominant (Fig. 2B). Rs5918762 is one of two transcribed members of the haplotype block represented by the tag SNP rs5919432 identified as a PC susceptibility risk locus in large genome‐wide association studies (Fig. 2C) [25, 26]. Of note, gV7_4 leading to the decreased expression of ARV7 in 22Rv1 (Fig. 1D,E), is in proximity to rs5918762, leading to the hypothesis that the allelic status of rs5918762 might affect gene expression. Indeed, when we genotyped rs5918762 in a previously characterized androgen deprivation therapy PC patient cohort consisting of specimens (*n* = 51) from trans‐urethral resections of the prostate (TURP) [13], a trend of decreased *ARV7* mRNA expression was measured in patients harboring the reference T allele (*P* = 0.0603, compared to alternative C allele), whereas *ARFL* mRNA levels were not affected by rs5918762 allelic state (Fig. 2D). To elucidate a molecular mechanism for this finding, RNAfold [27], an algorithm predicting secondary RNA structures, was interrogated with the full *ARV7* 3′UTR sequence. Results indicated that an additional loop may be present when the sequence contains the T allele compared to the C allele (Fig. S3A). Changes in the secondary or tertiary RNA structure may affect the binding of RNA‐binding proteins (RBP) that act on a post‐transcriptional level [3]. In particular, mRNA stability is regulated by the 3′UTR, postulating that rs5918762 C/T might change half‐life of *ARV7* mRNA. To corroborate this, we stably transduced the ARV7‐positive 22Rv1 cell line with N‐terminally Flag‐tagged ARV7 constructs containing the complete 3′UTR in its rs5918762 C or T allele and used a qPCR primer/probe combination to measure Flag expression by Roadblock‐qPCR mRNA decay assay [14]. However, only minor, statistically nonsignificant different half‐lives for ARV7 rs5918762 C (*T*_1/2_ = 2.673 h) vs. *T* (*T*_1/2_ = 3.267 h) were found (Fig. 2E). Furthermore, a dual luciferase assay conducted in the ARV7‐negative cell line HEK293FT using the 3′UTR of *ARV7* in different allelic states cloned 3′ to the firefly coding sequence showed no significant difference in luciferase activity in the alternative vs. reference allele for rs5918762 (Fig. S3B). In contrast, a decreased Firefly signal was measured for rs140982926 in the alternative allele, a rare SNP with a MAF of 0.003488%. These results let us conclude that the allelic status of rs5918762 might affect *ARV7* mRNA expression by other post‐transcriptional pathways than mRNA stability. Due to the rather short coding sequence of CE3 of 16 amino acids, exonic splicing enhancers/silencers might also be present in the proximal 3′UTR. To analyze a possible effect of rs5918762 on ARV7 splicing, we constructed a minigene assay with the two rs5918762 alleles, as depicted in Fig. 2F. Specific primer/probe pairs enable the detection of CE3 inclusion (ARV7 splicing) and CE3 exclusion (ARFL splicing) in the same sample. Indeed, an approximately 3‐fold increased CE3 inclusion rate was measured for rs5918762 C compared to T when the minigene was transfected to AR(V7)‐negative PC3 cells (Fig. 2G). Intriguingly, also an approximately 2‐fold increased CE3 exclusion rate was measured. Thus, we concluded that rs5918762 is involved in regulation of splicing of ARV7 leading to the decreased expression of *ARV7* mRNA when the SNP is in its T allele compared to the C allele.

### 3.3 Preferred binding of SRSF9 for rs5918762 C to promote ARV7 splicing

**Fig. 3:**
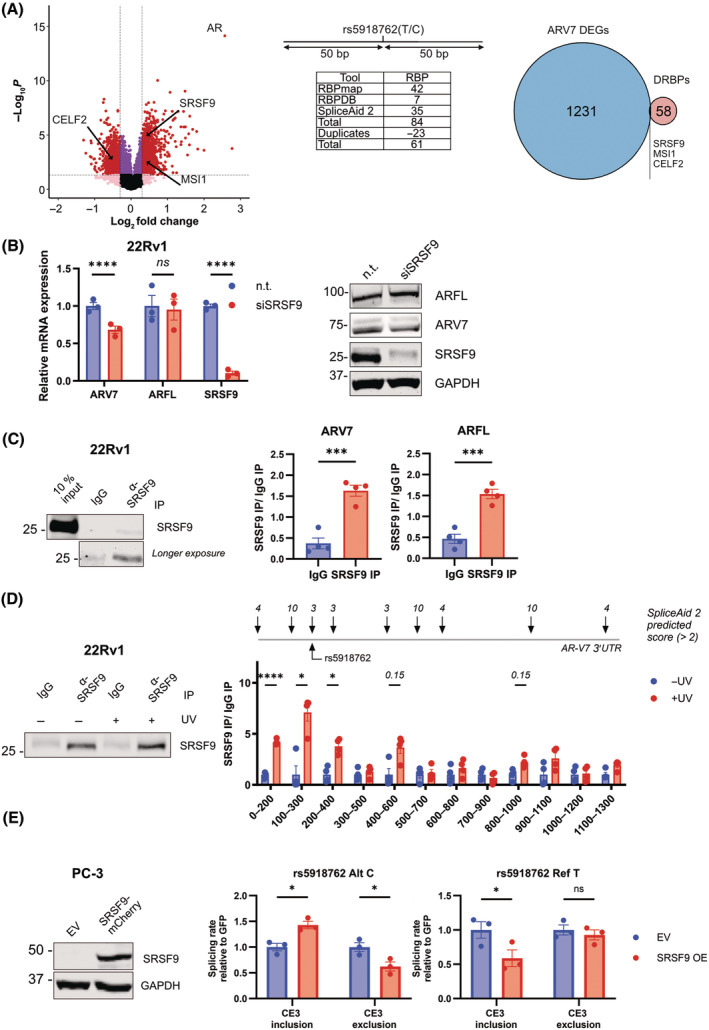
SRSF9 binds to ARV7's 3′UTR in the alternative rs5918762 C allele promoting CE3 inclusion. (A) Identification of SRSF9 as an RNA binding protein (RBP) for *ARV7*. (left) Differential gene expression (DEG) analysis based on *ARV7* expression cohorts (Q1 vs. Q4) with publicly available data from the SU2C/PCF consortium. (middle) *In silico* analysis of differentially binding RNA binding proteins (DRBPs). Three publicly available RBP predictors were queried with the *ARV7* 3′UTR sequence with rs5918762 in its C or T allele. Predicted rs5918762 differentially binding RBPs (DRBPs) were cleaned for duplicates. (right) Venn‐diagram depicting the intersection of differential gene expression analysis and DRBP analysis. (B) qPCR and immunoblotting of ARFL and ARV7 after SRSF9 downregulation. qPCR and immunoblot analyses were done after transfection of 22Rv1 with n.t. siRNA or a pool of 4 siRNAs specific for SRSF9. For qPCR analysis statistical analysis was done by Student's *t*‐test (mean ± SEM, *n* = 3, ****P < 0.0001). A representative blot is shown. Dot plots of relative protein expressions can be found in Fig. S4. (C) Binding analysis of SRSF9 to *ARV7/FL* mRNA by CLIP‐qPCR. The immunoblot (left) shows the specificity of the immunoprecipitation (IP). 15% (v/v) of the IP eluate were analyzed by immunoblotting, the remaining 85% were subjected to qPCR analysis (right). Statistical significance was calculated by Student's *t*‐test (mean ± SEM, *n* = 3, ***P < 0.001). (D) Fine‐mapping of SRSF9 binding on *ARV7*'s 3′ untranslated region (3′UTR) by CLIP‐qPCR. The experiment was repeated as in (C) with additional RNase T1 digestion prior immunoprecipitation. To confirm specific immunoprecipitation, RNA/RBP complexes were UV‐crosslinked or left untreated. Primer walking qPCR for the indicated amplicons was performed. On top, predicted binding scores (> 2) of SpliceAid 2 for SRSF9 are shown. Statistical significance was calculated by one‐way ANOVA (mean ± SEM, *n* = 3, *P < 0.05, ****P < 0.0001). (E) Impact of SRSF9 overexpression on the *ARV7* splicing ratio. PC‐3 cells were transfected with an overexpression plasmid for SRSF9‐mCherry or EV. Increased levels of SRFS9 are shown in a representative immunoblot. ARV7 minigene assay was performed for both alleles of rs5918762 after EV or SRSF9‐mCherry overexpression. Results were normalized for transfection efficiency by co‐transfection of GFP. Statistical significance was calculated by Student's *t*‐test (mean ± SEM, *n* = 3, ns = *P* > 0.05, *P < 0.05).

Next, we aimed to identify a causal RBP that has a preferred binding to *ARV7*'s 3′UTR in the rs5918762 C over T allele. To this end, we chose the following approach: using publicly available RNA sequencing datasets from 266 metastatic (m)PC specimen from the SU2C/PCF consortium [28, 29], a differential gene expression analysis of the highest and lowest quarter of *ARV7* spliced reads per million (SRPM) expression was done (Q1 vs. Q4, Fig. 3A, left). As expected, *AR* (describing all *AR* variants) was the most significantly upregulated gene and a total of 1231 genes were significantly deregulated. Next, we interrogated three available RBP predictors (RBP map [30], SpliceAid 2 [31], and RBPDB [32]) using the two different rs5918762 allelic states flanked by the respective 50 bp up‐ and downstream sequences. This resulted in a total of 61 predicted differentially binding RBPs (Fig. 3A, middle). The overlap of ARV7 and rs5918762 differentially binding proteins identified three candidates: SRSF9, MSI1, and CELF2 (Fig. 3A, right). SRSF9 is a member of the conserved serine/arginine (SR)‐rich family, being characterized as essential components of the spliceosome [33]. Since our previous results indicated that rs5918762 affects the splicing rate of *AR* pre‐mRNA (Fig. 2G), we decided to further investigate the role of the splicing factor SRSF9 in this process. Indeed, RNAi‐mediated downregulation of SRSF9 decreased ARV7 mRNA and protein expression in 22Rv1 demonstrating that SRSF9 is involved in the splicing process of ARV7 (Fig. 3B, Fig. S4). To prove the binding of SRSF9 to *ARV7* mRNA cross‐linking immunoprecipitation qPCR (CLIP‐qPCR) was performed. Although precipitation of SRSF9 by anti‐SRSF9 antibodies in UV‐crosslinked 22Rv1 (containing rs5918762 C) lysate was rather inefficient (see Section 2), a clear enrichment of *ARV7* mRNA, and also *ARFL* mRNA was measured after SRSF9 pull‐down compared to unspecific IgG precipitates (Fig. 3C). Fine‐mapping using CLIP‐qPCR combined with partial RNase digestion and primer‐walking qPCR revealed statistically significant binding peaks for SRSF9 on the proximal *ARV7* 3′UTR (amplicons: 0–400 bp) and trends of binding peaks in the middle and distal 3′UTR (amplicons: 400–600 bp, and 800–1100 bp), mirroring the predicted maximal SpliceAid 2 scores of 10 for SRSF9 (Fig. 3D). Of note, rs5918762 is located in a locus with predicted score of 3/10 in proximity to the first locus with score 10 prediction. Unfortunately, this adjacency does not allow the design of PCR primers to analyze the binding of SRSF9 to the rs5918762 locus independently of the score 10 locus. However, when repeating the minigene assay with the rs5918762 C allele in PC3 cells, overexpression of mCherry‐tagged SRSF9 was sufficient to further increase the CE3 inclusion rate compared to empty vector (EV) transfection (Fig. 3E). In contrast, using the minigene with the rs5918762 T allele a decreased CE3 inclusion was noted upon SRSF9 overexpression. Altogether this shows that SRSF9 is binding preferentially to *ARV7*'s 3′UTR in its rs5918762 C allele promoting an increased rate of ARV7 splicing.

### 3.4 Clinical relevance of CLK/SR proteins in PC

**Fig. 4:**
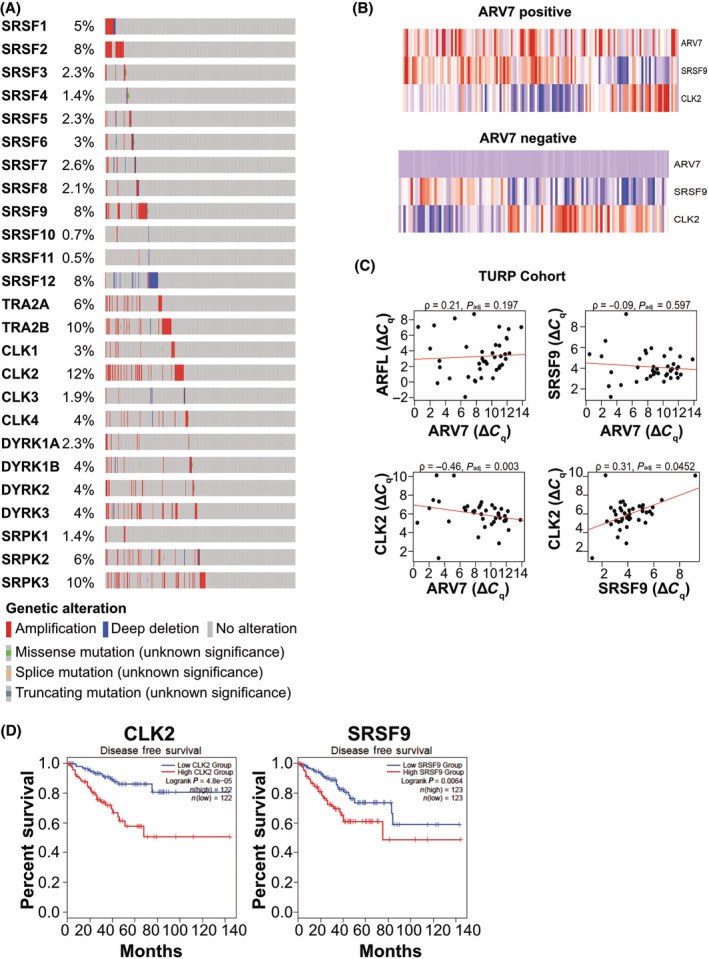
Increased expression of CLK2 or SRSF9 in metastatic PC correlates with bad prognosis. (A) Scheme depicting the copy number aberrations for the SR‐, CLK‐, DYRK‐, and SRPK‐protein families in metastatic PC patients from the SU2C/PCF database. Percentages of patients with copy number aberrations are indicated. (B) Gene expression of *ARV7*, *SRSF9* and *CLK2* in the SU2C/PCF cohort. Patients with RNA sequencing data were grouped in *ARV7*‐positive [> 0.5 splice reads per million (SRPM), top] and *ARV7*‐negative (< 0.5 SRPM, bottom) cohorts, which were clustered for *SRSF9* and *CLK2* expression. *ARV7* expression is shown on top, but was not included in clustering analysis. (C) Spearman correlation analysis of *ARFL*, *ARV7*, *CLK2*, and *SRSF9* gene expression in the trans‐urethral resection of the prostate (TURP) cohort (cf. Fig. 2D). Gene expression was measured by qPCR and correlation analyses were done. ρ indicates the strength and direction of correlation, *P* the statistical significance. (D) Kaplan–Meier plots of disease‐free survival stratified by *CLK2* (left) and *SRSF9* (right) mRNA expression using TCGA datasets. For analysis, the top and the bottom quarter of gene expression was used (Q1 vs. Q4). Statistical significance was calculated by the log rank test.

In general, the activity of the members of the SR family plays a critical role in the regulation of the pre‐mRNA processing machinery [34]. SR proteins are phosphorylated at multiple RS dipeptide repeats mainly by kinases of the CLK, DYRK and SRPK families. To assess the role of these families in alternative splicing in advanced PC, we screened for copy number aberrations in 444 specimens from publicly available whole exome sequencing data from the SU2C/PCF consortium [28, 29]. *CLK2* (11.3%) and *SRSF9* (7.4%) were found to be among the most amplified genes in their respective families, and 16.2% of all patients harbored either or both amplifications (Fig. 4A). In the SU2C/PCF dataset containing 266 RNA sequencing datasets an inverse correlation of SRSF9 and CLK2 expression was found in both *ARV7*‐positive and ‐negative patients (Fig. 4B). A higher percentage of patients with high *CLK2* expression was evident in the *ARV7*‐negative compared to the ‐positive cohort. Reanalysis of the TURP cohort used in Fig. 2D for *ARV7*, *ARFL*, *SRSF9* and *CLK2* expression by qPCR also showed a statistically significant negative correlation of *ARV7* with *CLK2*, however not with *SRSF9* (Fig. 4C). In contrast to the SU2C/PCF mPC dataset, the TURP cohort showed a positive correlation of *SRSF9* with *CLK2*. Moreover, Kaplan–Meier plots for disease‐free survival using data from the TCGA database [20] containing early stage PC demonstrated significantly decreased survival rates for high *CLK2* (Fig. 4D left) and high *SRSF9* (Fig. 4D, right) expression when the cohorts were chosen to contain the bottom and top quarter of the quantified *CLK2* or *SRSF9* reads, respectively. Altogether, this shows that CLK2 and SRSF9 have clinical relevance correlated with alternative splicing of *AR* in late stage PC, leading us to hypothesize that a direct or indirect interaction between CLK2 and SRSF9 regulating this process may occur.

### 3.5 Regulation of ARV7 expression by the CLK2/SRSF9 axis

**Fig. 5:**
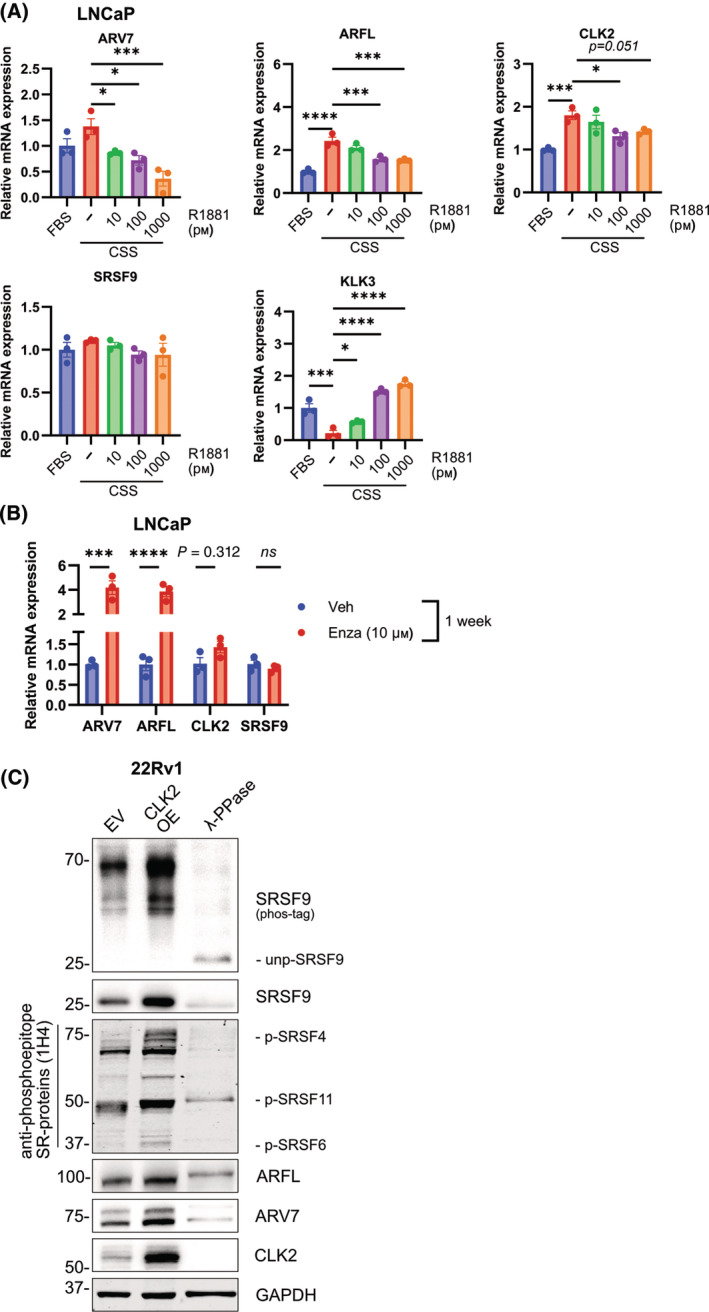
The AR regulates its own splicing by interfering with the CLK2/SRSF9 axis. (A, B) Androgenic regulation of CLK2. (A) Castration‐sensitive LNCaP were cultured for 1 week under steroid‐deprived conditions using charcoal‐stripped serum (CSS) or normal FBS, as indicated. Treatments with increasing concentrations (10–1000 pm) of the synthetic androgen R1881 was done for the last 24 h. qPCR was performed for ARV7, ARFL, CLK2, SRSF9, and as control KLK3 encoding PSA. Statistical testing was done by Student's *t*‐test (mean ± SEM, *n* = 3, *P < 0.05, ***P < 0.001, ****P < 0.0001). (B) qPCR for ARFL, ARV7, CLK2, and SRSF9 of LNCaP cells cultured under normal growth conditions with the addition of 10 μm Enzalutamide (Enza) or vehicle (veh). Statistical testing was done by Student's *t*‐test (mean ± SEM, *n* = 3, ns = *P* > 0.05, ***P < 0.001, ****P < 0.0001). (C) Protein expression analysis of 22Rv1 stably overexpressing CLK2 (CLK2 OE) for total SRSF9, phosphorylated SR proteins, ARFL and ARV7. Identification of the indicated phosphorylated SR proteins was based on their molecular weight. The same samples were loaded in parallel on a phos‐tag gel and probed with the SRSF9 antibody to analyze its phosphorylation status. As control for the phosphorylation degree, a sample was treated with lambda protein phosphatase (λ‐PPase) prior loading on the gel. unp‐SRSF9, unphosphorylated SRSF9. A representative blot is shown (*n* = 3). Dot plots of relative protein expressions and of the ratio p‐SRSF9/SRSF9 can be found in Fig. S5A.

Previously, AR‐binding sites found within the 300 kb region upstream of the transcription start site of the *CLK2* gene [35] have been associated with an AR‐mediated downregulation of *CLK2* [36]. We have confirmed these results with the castration‐sensitive LNCaP cell line expressing low ARV7 mRNA levels [37]. Steroid‐deprivation (Fig. 5A) or AR inhibition by Enza (Fig. 5B) for 7 days each was sufficient to increase CLK2 [1.8×; 1.4× (not significant)], ARFL (2.4×; 3.9×) and ARV7 (1.4×; 4.2×), respectively. Furthermore, reactivation of ARFL's function by supplementation with the synthetic androgen R1881 in steroid‐starved LNCaP was able to reverse the effect on *CLK2* expression at higher concentrations, while an androgenic regulation of SRSF9 was not observed (Fig. 5A). Combined with the observed inverse correlation of *CLK2* and *SRSF9* expression (Fig. 4B), androgenic regulation of CLK2 may represent a possible regulation of the spliceosome and thus of alternative *AR* splicing. To elaborate a possible link between CLK2 and SRSF9, we set out to analyze the consequences of modulation of CLK2 expression. To this end, 22Rv1 cells stably overexpressing CLK2 via lentiviral transduction were generated. Increased activity of CLK2 was evidenced by the anti‐phosphoepitope SR proteins antibody showing the increased phosphorylation of a number of SR proteins (Fig. 5C). Analysis of phosphorylated SRSF9 by phos‐tag blots in 22Rv1‐CLK2 showed no increase in the ratio of p‐SRSF9/total SRSF9, however a clear increase in total SRSF9 levels was apparent (Fig. 5C, Fig. S5A). Importantly, CLK2 overexpression resulted in increased expression of ARV7, while a positive trend for ARFL expression was observed. Of note, SRSF9 mRNA levels were unchanged after CLK2 overexpression, indicating a (post)‐translational mechanism of CLK2 on SRSF9 protein expression (Fig. S5B). A regulatory feedback mechanism involving CLK2 could be excluded, since SRSF9 knockdown did not affect CLK2 expression levels significantly (Fig. S5D). In summary, we concluded that inhibition of AR activity leads to increased *CLK2* expression which in turn leads to increased SRSF9 protein levels favoring splicing of ARV7.

### 3.6 Pharmacological inhibition of CLK2 leads to the decreased ARV7 expression

**Fig. 6:**
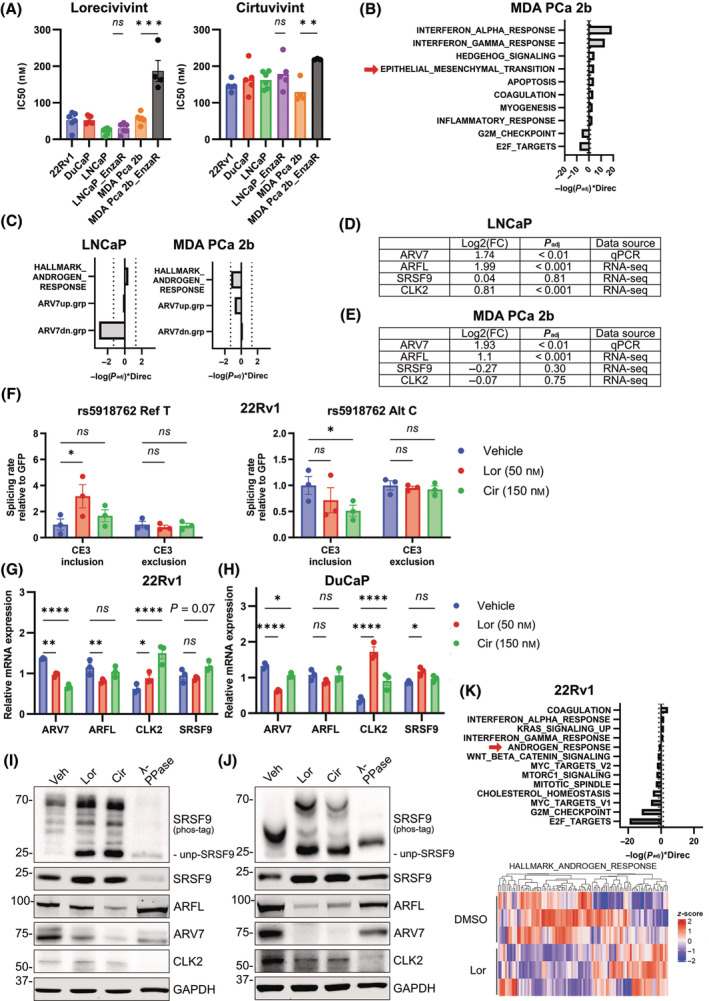
Treatment with the splicing inhibitors Lor and Cir leads to decreased ARV7 expression. (A) IC50 determinations for Lorecivivint (Lor) and Cirtuvivint (Cir) in a panel of PC cell lines. Indicated cell lines were treated with increasing concentration of both inhibitors and live cell imaging with confluency measurements (Incucyte) was done. IC50 concentrations were calculated for each cell line by dose response curves and statistical significance between cell lines was calculated by Student's *t*‐test (mean ± SEM, *n* = 3, ns = *P* > 0.05, **P < 0.01, ***P < 0.001). (B) Differential gene expression of MDA PCa 2b Enzalutamide resistant (EnzaR) vs. MDA PCa 2b cells profiled for gene set enrichment analysis using the Hallmark pathways from RNA sequencing data (*n* = 3). Red arrow indicates Hallmark pathway “Epithelial Mesenchymal Transition”. (C) Detailed depiction of the gene set enrichment analysis of the Hallmark pathway “Androgen response” and the specific ARV7 gene set (up: upregulated genes, down: downregulated genes) for the two LNCaP (left) and MDA PCa 2b (right) ‐EnzaR/parental pairs from RNA sequencing data (*n* = 3). Dashed lines indicate significance thresholds of *P* = 0.05. (D, E) Tables showing log fold changes (Log2 FC) of *ARFL*, *ARV7*, *CLK2* and *SRSF9* in LNCaP‐EnzaR vs. LNCaP (D) and MDA PCa 2b‐EnzaR vs MDA PCa 2b (E) as assessed by RNA sequencing or qPCR (*n* = 3, statistical test used: Student's *t*‐test). (F) ARV7 splicing rate assessed with the minigene assay for rs5918762 C or T allele after treatment with Lor or Cir. ARV7 minigene assay was repeated as in Fig. 2G after treatment with the IC50 concentrations of Lor (50 nm), Cir (150 nm), or vehicle (DMSO) and statistical significance was assessed by one‐way ANOVA (mean ± SEM, *n* = 3, ns = *P* > 0.05, *P < 0.05). (G–J) Expression levels of ARFL, ARV7, CLK2, SRSF9 and phosphorylation level of p‐SRSF9 after treatment with Lor or Cir on mRNA level (22Rv1, G; DuCaP, H) and protein level (22Rv1, I; DuCaP, J). Phosphorylation of SRSF9 was assessed by phos‐tag immunoblotting. (I, J) To assess dephosphorylation of SRSF9, a sample was treated with lambda protein phosphatase (λ‐PPase) prior loading on the gel. unp‐SRSF9, unphosphorylated SRSF9. A representative blot is shown. Dot plots of relative protein expressions can be found in Fig. S6E. (G, H) Statistical significance was assessed by one‐way ANOVA (mean ± SEM, *n* = 3, ns = *P* > 0.05, *P < 0.05, **P < 0.01, ****P < 0.0001). (K) (left) Differential gene expression of 22Rv1 treated with 50 nm Lor vs. untreated (DMSO) profiled for gene set enrichment analysis using the Hallmark pathways. Red arrow indicates Hallmark pathway “Androgen Response”. (right) Heatmap of differential expressed genes of the Hallmark pathway “Androgen response”. Dashed lines indicate significance thresholds of *P* = 0.05 (*n* = 3).

The CLK family is of vast interest for a number of diseases and numerous inhibitors against members of this family have been developed [34]. Based on our findings, we sought to analyze the efficiency of these inhibitors in PC models. To this end, we selected two compounds with different characteristics: Cirtuvivint (SM08502, Cir) [38] a pan‐CLK/DYRK inhibitor currently undergoing phase I clinical trials in patients with solid tumors including PC (clinicaltrials.gov: NCT03355066, NCT05084859), and Lorecivivint (SM04690, Lor) [39], an inhibitor with highest affinity for CLK2 [40] with finished phase III trials for osteoarthritis of the knee (e.g. NCT04520607). Besides the ARV7‐expressing cell lines 22Rv1 and DuCaP, we also included for our preclinical studies two cell line pairs (LNCaP and MDA PCa 2b) that acquired resistance to Enza (−EnzaR) by continuous treatment with the drug, as depicted in Fig. S6A. In line with previous studies [40], both small molecules were potent inhibitors of cell growth at nanomolar concentrations for all included cell lines (Fig. 6A). Cir was effective in inhibiting proliferation by 50% at concentrations of approximately 150 nm, while Lor was more potent with an IC50 at approximately 50 nm already. Interestingly, MDA PCa 2b‐EnzaR had a significantly lowered sensitivity to both compounds, which was not the case for LNCaP‐EnzaR. To find a molecular explanation for this discrepancy, we performed transcriptome analysis by RNA sequencing, differential gene expression, and pathway analysis for both EnzaR derivatives compared to their parental cell lines, respectively. Bioinformatic analyses for LNCaP have been published previously [10], for MDA PCa 2b results are depicted in Fig. 6B, Fig. S6B, and in Table S2. Of note, LNCaP cells harbor the rs5918762 alternative C allele, while MDA PCa 2b cells originating from an African American patient have the reference T allele (Fig. S6C) [41]. Gene set enrichment analysis (GSEA) using a previously published gene set specific for ARV7 [42] evidenced transcriptional activity of ARV7 in LNCaP‐EnzaR (Fig. 6C, left). This was in line with statistically significant increases in *ARFL*, *ARV7* and *CLK2* (Fig. 6D), showing that a re‐activation of the AR signaling axis through ARV7 is associated with Enza resistance in this cellular model. In contrast, the MDA PCa 2b‐EnzaR transcriptome was enriched for genes involved in epithelial‐to‐mesenchymal transition (EMT, Fig. 6B), which was concordant with a changed morphology of these cells (Fig. S6D). Interestingly, ARFL and ARV7 expression in MDA PCa 2b‐EnzaR were also significantly increased (Fig. 6E), however no statistically significant enrichment for an ARV7‐specific signature, or for the hallmark pathway Androgen Response was detected (Fig. 6C, right). Next, we were interested in an rs5918762 allele‐specific effect of Lor and Cir for ARV7 splicing. Repetition of the minigene assay in 22Rv1 after treatment with IC50 concentrations of Lor and Cir revealed a decreased CE3 inclusion rate for the C allele for Cir, while an increased rate for the T allele for Lor was measured (Fig. 6F). Of note, ARFL splicing (CE3 exclusion) was not altered in either allele. This confirms that this SNP is an important determinant in the *AR* pre‐mRNA splicing process. Furthermore, we analyzed the effects of both compounds on the mRNA and protein levels of ARFL, ARV7, CLK2, SRSF9, and the phosphorylation status of SRSF9 in 22Rv1 and DuCaP (Fig. 6G–J, Fig. S6E). Both compounds at IC50 concentrations decreased protein expression of both ARFL and ARV7. While also *ARV7* mRNA was decreased upon treatment with the inhibitors, less significant effects could be observed on *ARFL* mRNA, postulating that also (post‐)translational mechanisms are affected by Lor and Cir treatment. *CLK2* mRNA expression was significantly increased on mRNA level, whereas on protein level no consistent change in expression was observed. Interestingly, SRSF9 expression was increased by both compounds, which could be attributed to accumulation of newly formed SRSF9 remaining in an unphosphorylated state due to the action of the inhibitors or be the result of dephosphorylation of p‐SRSF9. Strikingly, only a minor fraction of unphosphorylated SRSF9 could be detected in the controls, suggesting that both cell lines rely on active SRSF9 for its alternative splicing. Presumably, inhibition of CLK2 leading to a disturbed spliceosome will not only affect splicing of AR, but also other signaling pathways. We were therefore interested to assess the relative specificity of Lor for AR splicing in the 22Rv1 cell line using a combined approach of short‐read (Fig. 6K, Fig. S6F, Table S3) and long‐read, isoform‐specific RNA sequencing (Fig. S6G, Table S4). Pathway analysis using short‐read sequencing revealed that the hallmark Androgen Response was in the top down‐regulated pathways (Fig. 6K), among other PC‐relevant oncogenic pathways, i.e. MYC, cell cycle control (E2F Targets and G2M Checkpoint), and as previously published [39], WNT/β‐catenin (15th most significant, see Table S3). Pathway analysis on the gene level of the long‐read RNA sequencing dataset revealed a similar set of deregulated pathways upon Lor treatment (Fig. S6G). Altogether, we conclude that inhibition of the CLK/DYRK family by Cir and Lor, results in a decreased phosphorylation of SRSF9, likely yielding a disturbed splicing machinery and concomitantly in a decreased expression of ARFL and ARV7, which, among other signaling pathways, contributes to the inhibitors' effects on cellular proliferation of Enza‐sensitive and ‐resistant PC cell lines.

### 3.7 Combination of Enza with Lor has additive effects on cellular proliferation

**Fig. 7:**
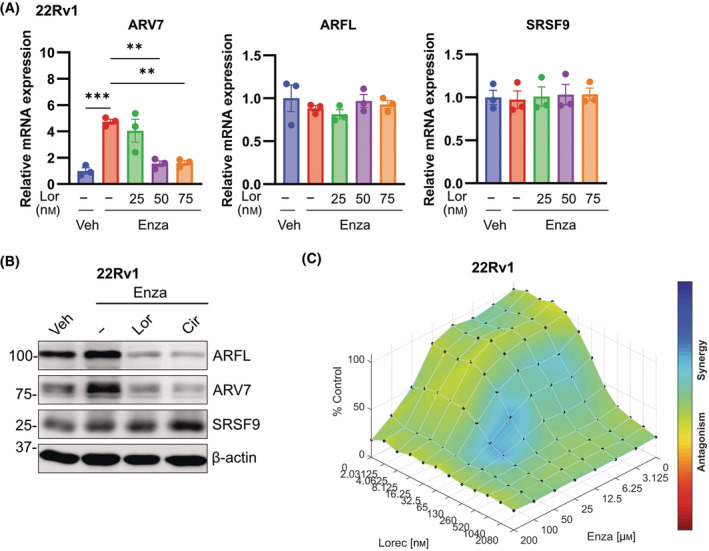
CLK2 inhibition results in sensitization to Enzalutamide. (A) Dose‐dependent decrease of *ARV7* mRNA in Enzalutamide (Enza) pretreated cells after Lorecivivint (Lor) treatment. 22Rv1 were pre‐treated with 10 μm Enza or vehicle (EtOH) for 7 days and then additionally treated with 25, 50, and 75 nm Lor or vehicle (DMSO) for 3 days. qPCR was then performed for *ARV7*, *ARFL*, and *SRSF9*. Statistical significance was assessed by one‐way ANOVA (mean ± SEM, *n* = 3, **P < 0.01, ***P < 0.001). (B) Decrease of ARFL and ARV7 protein in Enza‐pretreated cells after Lor or Cirtuvivint (Cir) treatment. Experiment was repeated as in (A), using the IC50 concentrations for Lor (50 nm) and Cir (150 nm). A representative blot is shown (*n* = 3). Dot plots of relative protein expressions can be found in Fig. S7. (C) Synergy analysis of Enza and Lor co‐treatment in 22Rv1. Incucyte confluency measurements were done after treating 22Rv1 with increasing doses of Lor and Enza for 3 days (*n* = 3). Drug synergism was analyzed using combenefit software and its Highest Single Agent (HSA) model.

Overexpression of CLK2 (Fig. 5C) or inhibition of CLK2 by Lor and Cir (Fig. 6I,J) altered ARV7 expression via a mechanism involving SRSF9. We were therefore interested whether disturbing the CLK2/SRSF9 axis by CLK2 inhibition might result in additive effects on cellular proliferation after Enza treatment. Thus, we assessed the dose‐dependent effect of Lor in 22Rv1 after pre‐treatment for 7 days with 10 μm Enza. Enza treatment led to increased expression of *ARV7* mRNA, while no significant changes were observed for *ARFL* mRNA (Fig. 7A). Lor at the IC50 concentration was able to decrease *ARV7* mRNA expression levels to almost basal, non‐Enza treated levels. Similar effects were observed on the protein level, where Enza pre‐treatment increased the expression of ARFL and ARV7 (Fig. 7B, Fig. S7). Both compounds decreased the expression of ARFL and ARV7 protein, confirming the results of Fig. 6I,J. Finally, a HSA synergism analysis [43] using live cell imaging proliferation assays with increasing concentrations of Lor and Enza in 22Rv1 was done (Fig. 7C). Additive effects for both compounds were found with slight synergism at higher concentrations of both drugs. In summary, we concluded that a combination therapy of Enza and Lor is inhibiting or decreasing expression of ARFL and ARV7 isoforms, respectively, leading to decreased cellular growth. However, a contribution of other Lor‐affected oncogenic pathways (Fig. 6K) to the observed efficiency of the combination therapy must be taken into account.

## 4 Discussion

### 4.1 Function of SRSF9 in the regulation of ARV7 expression

ARSI are widely used for the treatment of CRPC, increasingly also in the castration‐sensitive stage of the disease. Increased expression of ARV7 is one of the molecular changes caused by AR blockade with 75% of all patients expressing ARV7 following androgen‐deprivation therapy and further increases after treatment with ARSI [44]. Since ARV7 arises via aberrant alternative splicing of the AR gene [1], targeting of this process might be an approach to inhibit ARV7's oncogenic action. Within this concept, the fact of *ARFL* and *ARV7* having distinct 3′UTRs could be exploited to specifically target ARV7. Here, we could demonstrate that *ARV7*'s 3′UTR is a regulator of its splicing process through the binding of the splicing factor SRSF9 to the proximal 3′UTR. CE3 codes only for 16 amino acids corresponding to 51 nucleotides (including the stop codon), which may explain the binding of splicing factors to the 3′UTR rather than to the coding sequence. In general, the binding of SR‐proteins to 3′UTRs has been documented previously. Their functions included e.g. inhibition of mRNA translation (SRSF3 binding to the 3′UTR of p21^cip1/waf1^) [45] or length modification of the 3′UTR (SRSF3/7 binding to proximal adenylation sites) [46]. In case of *ARV7* mRNA, the binding of SRSF1 to an exonic splicing enhancer was mapped to a region overlapping with the stop codon of ARV7 [7], while the SRSF3 binding site was not determined [8].

Using a minigene assay, we were able to show that SRSF9 overexpression changes the CE3 inclusion rate, however the exact molecular mechanism of SRSF9 in this process remains unclear. In a recent study, SRSF9 was found to bind to mRNA in a N6‐Methyladenosine (m6A)‐dependent manner, thereby decreasing mRNA stability [47]. However, for *ARV7* mRNA, changing the allelic state of rs5918762 from the alternative C to reference T did not impact *ARV7* mRNA decay rate (Fig. 2E), but on the other hand this was sufficient to alter the CE3 inclusion rate after the overexpression of SRSF9 (Fig. 3E). Thus, an involvement of SRSF9 in *ARV7* mRNA stability is unlikely. Interestingly, SRSF9 binding to the DNA‐ and RNA‐associated Y‐box protein 1 (YB1) resulted in YB1 nuclear accumulation, promoting the selection of alternative splice sites [48]. However, we were not successful in demonstrating the binding of SRSF9 to ARV7 protein (data not shown), ruling out a regulation of (its own) splicing through a SRSF9/ARV7 protein complex. Conversely, SRSF9 was also characterized for repressing 3′ splice site utilization [49] showing that SRSF9's action in the splicing process might be site‐ and context‐selective and possibly involve other factors. Thus, further studies clarifying the exact role of SRSF9, and of other SR‐proteins, in *AR* pre‐mRNA processing are in need.

### 4.2 Regulation of ARV7's splicing process through CLK2

The CLK family is well known to regulate the splicing process, and in particular alternative splicing, by phosphorylating the SR family members at their consensus R‐x‐x‐S/T sequence [34]. Tight control of CLK activity is fine‐tuning the splicing process in response to physiological changes, which can e.g. be observed by the body temperature‐sensitive regulation of CLK activity and thus SR‐mediated splicing [50]. This need for tight control of CLK2 abundance and activity can also be observed after exogenous overexpression of active CLK2, which led to increased SRSF9 and ARV7 expression levels (Fig. 5C). Interesting in this context is also the negative correlation of *CLK2* and *SRSF9* mRNA expression in the mPC cohort (Fig. 4B), and that CLK2 regulates SRSF9 expression rather than its phosphorylation degree in the 22Rv1 cell line. This suggests that SRSF9 splicing activity can either be adjusted post‐translationally via a mechanism involving CLK2 and that further studies to elucidate the exact mechanism of the CLK2/SRSF9/ARV7 axis are warranted. Furthermore, other factors, such as SRSF1, which is regulating ARV7 splicing [7] and which is activated by CLK2 [51] may be involved.

### 4.3 Implications of rs5918762's contribution to ARV7 splicing

In our cohort of 51 TURP specimens we were able to detect a trend (*P* = 0.0603) of decreased *ARV7* mRNA expression, when rs5918762 was in its T compared to the C allele (Fig. 2D). In general, advanced PC specimens are difficult to obtain, as surgery in these stages is only infrequently performed. Palliative TURP is usually done in men with PC and urinary tract problems [52]. Despite these problems, following up on genotyping more advanced PC specimens might lead to the detection of a novel biomarker to discriminate between ARV7^high^ and ARV7^low^‐positive patients. Such a predictive biomarker would be useful for stratifying the therapeutic regimen (taxanes vs. ARSI) of patients. Compared to the existing test, which detects nuclear ARV7 protein in circulating tumor cells [2], rs5918762 genotyping of patients' nontumor cells is less labor‐ and cost‐intensive.

Worth mentioning is also the fact that rs5918762 belongs to the haplotype block represented by the tag SNP rs5919432, which was identified as PC susceptibility risk locus [25, 26]. At first view, an involvement of this SNP in prostate carcinogenesis is not obvious, as primary, treatment‐naive PC specimens only rarely express ARV7 [44]. However, the situation might be different in the rare prostate tumor‐initiating cells, which have been identified as luminal progenitors resistant against castration [53]. It can be speculated that these cells with stem cell‐like characteristics may have an alternative splicing pattern, similar to tissue stem cells [54]. In this scenario, alternative splicing in luminal progenitors might upregulate ARV7 in an rs5918762‐dependent way, thereby contributing to the increased risk of developing cancer. Novel isoform‐specific, single cell RNA sequencing techniques might be able to address this hypothesis [55].

### 4.4 Therapeutic targeting of CLKs

SRSF9 was previously identified as a proto‐oncogene for its role as a regulator of WNT‐signaling [56, 57], the latter also being a well‐characterized pathway in PC [58]. However, specific inhibitors for SR proteins are currently not available, as this family has been recognized to have intrinsically disordered structures [59]. Splice‐switching oligonucleotides [60] that specifically bind to the SR recognition site on the *AR* pre‐mRNA could be considered to downregulate ARV7 without disturbing the general splicing machinery [61]. On the other hand, a number of inhibitors against the SR‐upstream kinases CLK/DYRK are available [40]. We were able to show that CLK2 is affecting SRSF9 expression (Fig. 5C) and that its inhibition through CLK/DYRK inhibitors resulted in a decreased AR signaling response (Fig. 6K). In both short (15th most significant) and long‐read transcriptomic (8th most significant) analyses we were also able to detect downregulation of the WNT/ꞵ‐Catenin pathway on the gene level after treatment with Lor (Fig. S6G, Tables S3 and S4). Another important signaling pathway driving aggressive PC, MYC [62], was also shut down in Lor‐treated cells. This shows that besides the AR pathway, other oncogenic pathways are affected after targeting the splicing machinery through CLK/DYRK inhibition.

Both inhibitors are currently undergoing clinical trials. Albeit we found Lor to be efficient at lower concentrations (Fig. 6A), both compounds showed similar effects on downregulation of ARFL and ARV7 (Fig. 6G–J). In *in vitro* protein kinase assays, Lor has an IC50 of 13.6 nm for CLK2, 76.1 nm for CLK3, 48.0 nm for DYRK1A and 89.0 nm for DYRK1B [40]. Lor is applied intra‐articular and considered safe and well‐tolerated with no significant side effects [63]. However, no data for pharmacodynamics, ‐kinetics, safety and tolerability is available for oral or intravenous (systemic) application. Cir is an orally available small molecule and well‐tolerated in pre‐clinical xenograft studies with acceptable body weight changes (< 10%) [38]. Cir has high inhibiting capacity among the CLK/DYRK family with IC50 values (*in vitro* kinase assays) ranging between 3.6 nm (for CLK2) and 86.4 nm (for DYRK4), except for DYRK3 (2.1 μm) [40]. A combination of Cir/Lor with ARSI might be a valuable approach to improve the current endocrine treatment regimens in CRPC. Such a combination treatment would have the advantage of fully blocking the AR axis, i.e. ARFL by Enza and ARV7 by Cir/Lor through interfering with *ARV7*'s splicing process. Collectively, Lor and Cir are interesting drug candidates for the treatment of ARV7‐positive cancer, albeit the specificity is not limited to the AR pathway.

## 5 Conclusions

In conclusion, we were able to identify SRSF9 as factor binding to *ARV7*'s 3′UTR and regulating its splicing. The splicing efficiency was dependent on the allelic status of the SNP rs5918762 located in the proximal 3′UTR. In addition, we found that CLK2 is involved in the regulation of SRSF9's expression, thereby mediating the expression of ARV7. Lor and Cir, two clinically investigated inhibitors of CLK2 and other CLKs/DYRKs have anti‐cancer activity in PC cell lines and lead to ARFL/ARV7 protein downregulation. A combination of Lor with Enza showed additive effects on reducing PC cell growth. This shows that targeting alternative splicing in advanced PC is a possible approach to improve the current treatment regimens.

## Conflict of interest

The authors declare no conflict of interest.

## Author contributions

JVG contributed to data acquisition, analysis and interpretation; project design; figure assembly; and manuscript writing. MP contributed to data acquisition, analysis and interpretation. FH contributed to bioinformatic analyses. EM and AK contributed to data acquisition and analysis. OS contributed to funding acquisition, providing material; data acquisition, analysis and interpretation. GK contributed to providing material; data acquisition, analysis and interpretation. MVC contributed to funding acquisition; conception. FRS contributed to funding acquisition; data acquisition, analysis and interpretation; conception; manuscript writing; supervision. All authors contributed to manuscript proofreading and final approval.

### Peer review

The peer review history for this article is available at https://www.webofscience.com/api/gateway/wos/peer‐review/10.1002/1878‐0261.13728.

## Supporting information


**Fig. S1.** Uncropped immunoblots of this study.
**Fig. S2.** Supporting data for Fig. 1: An intact 3′UTR sequence is important for ARV7 mRNA expression.
**Fig. S3.** Supporting data for Fig. 2: rs5918762 is a common SNP located in the 3′UTR of ARV7 having a role in AR alternative splicing.
**Fig. S4.** Supporting data for Fig. 3: SRSF9 binds to ARV7's 3′UTR in the alternative rs5918762 C allele promoting CE3 inclusion.
**Fig. S5.** Supporting data for Fig. 5: The AR regulates its own splicing by interfering with the CLK2/SRSF9 axis.
**Fig. S6.** Supporting data for Fig. 6: Treatment with the splicing inhibitors Lor and Cir leads to decreased ARV7 expression.
**Fig. S7.** Supporting data for Fig. 7: CLK2 inhibition results in sensitization to Enzalutamide.

**Table S1.** Characteristics of the patient cohort.

**Table S2.** Results of bioinformatical analysis of Fig. 6B,C.

**Table S3.** Results of bioinformatical analysis of Fig. 6K.

**Table S4.** Results of bioinformatical analysis of Fig. S6F,G.

**Table S5.** List of materials used in this study.

**Table S6.** Raw data of all plots of this study.

## Data Availability

(Raw) data collected in this study will be made available to researchers upon reasonable request to the corresponding author. RNA sequencing datasets (.fastq files) are available via the Gene Expression Omnibus (GEO) database under the following accession numbers: GSE254612 (differential gene expression MDA PCa 2b‐EnzaR vs. MDA PCa 2b); GSE254611 (differential gene expression 22Rv1 IC50 Lor vs. 22Rv1 vehicle, short‐read sequencing); GSE259334 (differential gene expression 22Rv1 IC50 Lor vs. 22Rv1 vehicle, long‐read sequencing). Materials used in this study, supplementary data (uncropped immunoblots), and raw data of all plots can be found in Table S5, Fig. S1, and Table S6, respectively.
