# Supplementary material for: Targeting the CLK2/SRSF9 splicing axis in prostate cancer leads to decreased ARV7 expression
Source: Mol Oncol. 2024 Sep 11;19(2):496–518. doi: 10.1002/1878-0261.13728 (PMC11792998; doi:10.1002/1878-0261.13728)
Supplement: Supplementary file 1 — Fig. S1. Uncropped immunoblots of this study. Fig. S2. Supporting data for Fig. 1: An intact 3′UTR sequence is important for ARV7 mRNA expression. Fig. S3. Supporting data for Fig. 2: rs5918762 is a common SNP located in the 3′UTR of ARV7 having a role in AR alternative splicing. Fig. S4. Supporting data for Fig. 3: SRSF9 binds to ARV7's 3′UTR in the alternative rs5918762 C allele promoting CE3 inclusion. Fig. S5. Supporting data for Fig. 5: The AR regulates its own splicing by interfering with the CLK2/SRSF9 axis. Fig. S6. Supporting data for Fig. 6: Treatment with the splicing inhibitors Lor and Cir leads to decreased ARV7 expression. Fig. S7. Supporting data for Fig. 7: CLK2 inhibition results in sensitization to Enzalutamide. [file MOL2-19-496-s004.zip › mol213728-sup-0001-FigS1.pdf]

Uncropped immunoblots

Supplementary Figure 1, Van Goubergen et al., 2024

Fig. 1E

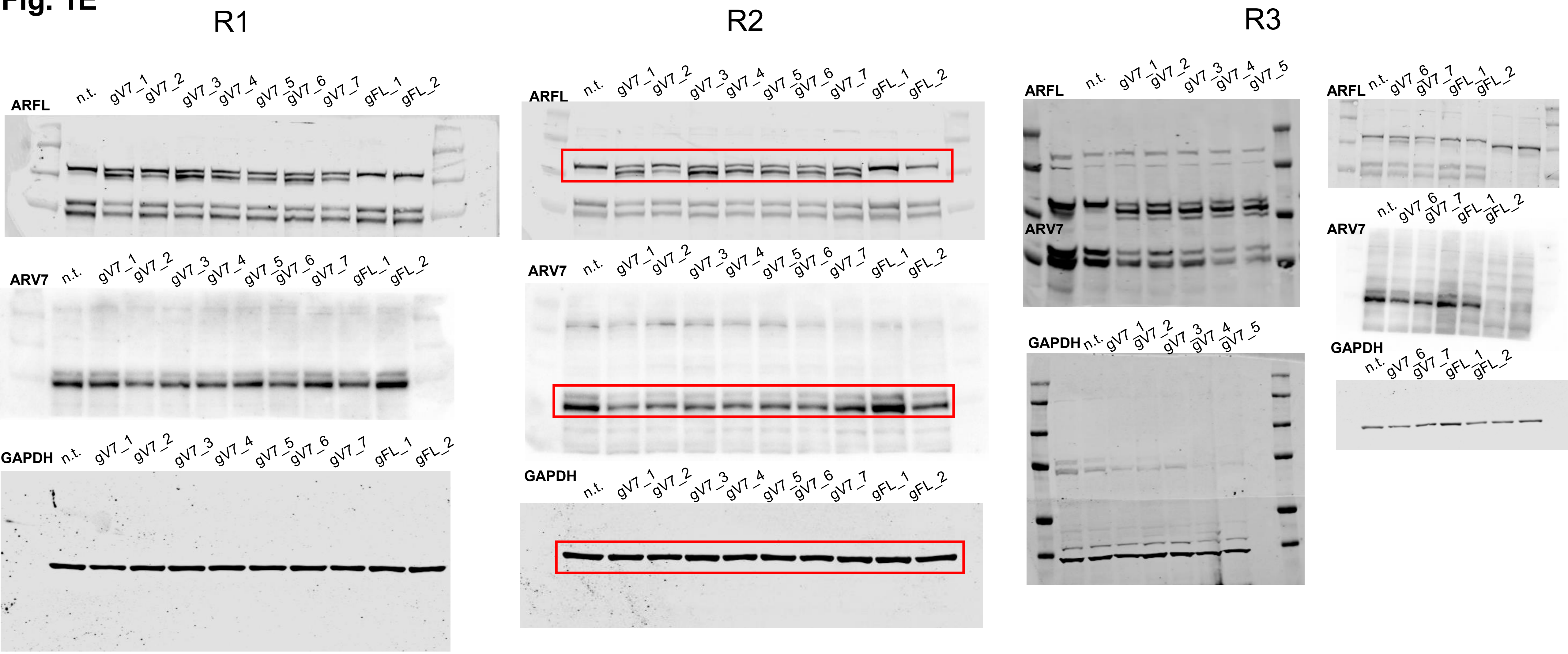
R1
R2
R3
ARFL
ARV7
GAPDH
n.t.
gV7_1
gV7_2
gV7_3
gV7_4
gV7_5
gV7_6
gV7_7
gFL_1
gFL_2

Fig. 1G

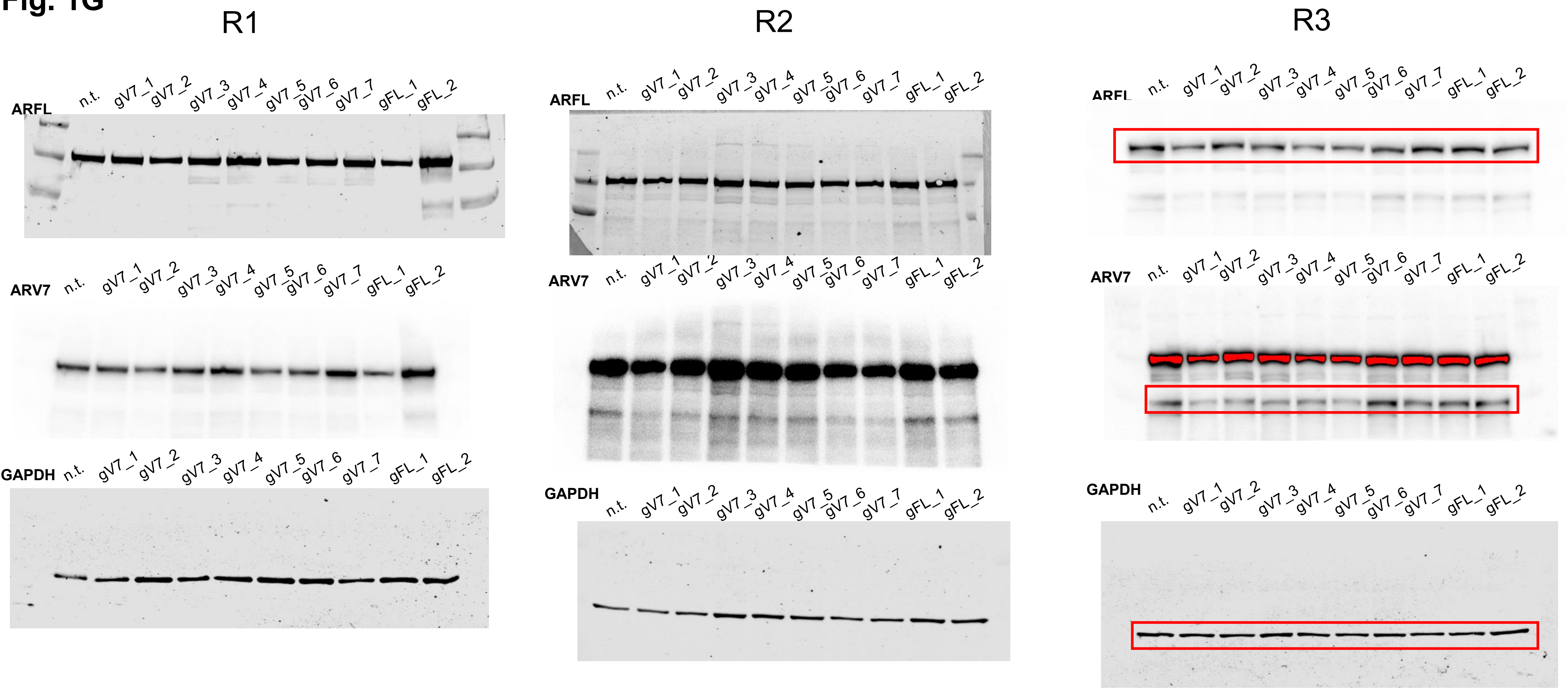
R1
R2
R3
ARFL
ARV7
GAPDH
n.t.
gV7_1
gV7_2
gV7_3
gV7_4
gV7_5
gV7_6
gV7_7
gFL_1
gFL_2

Fig. 3B

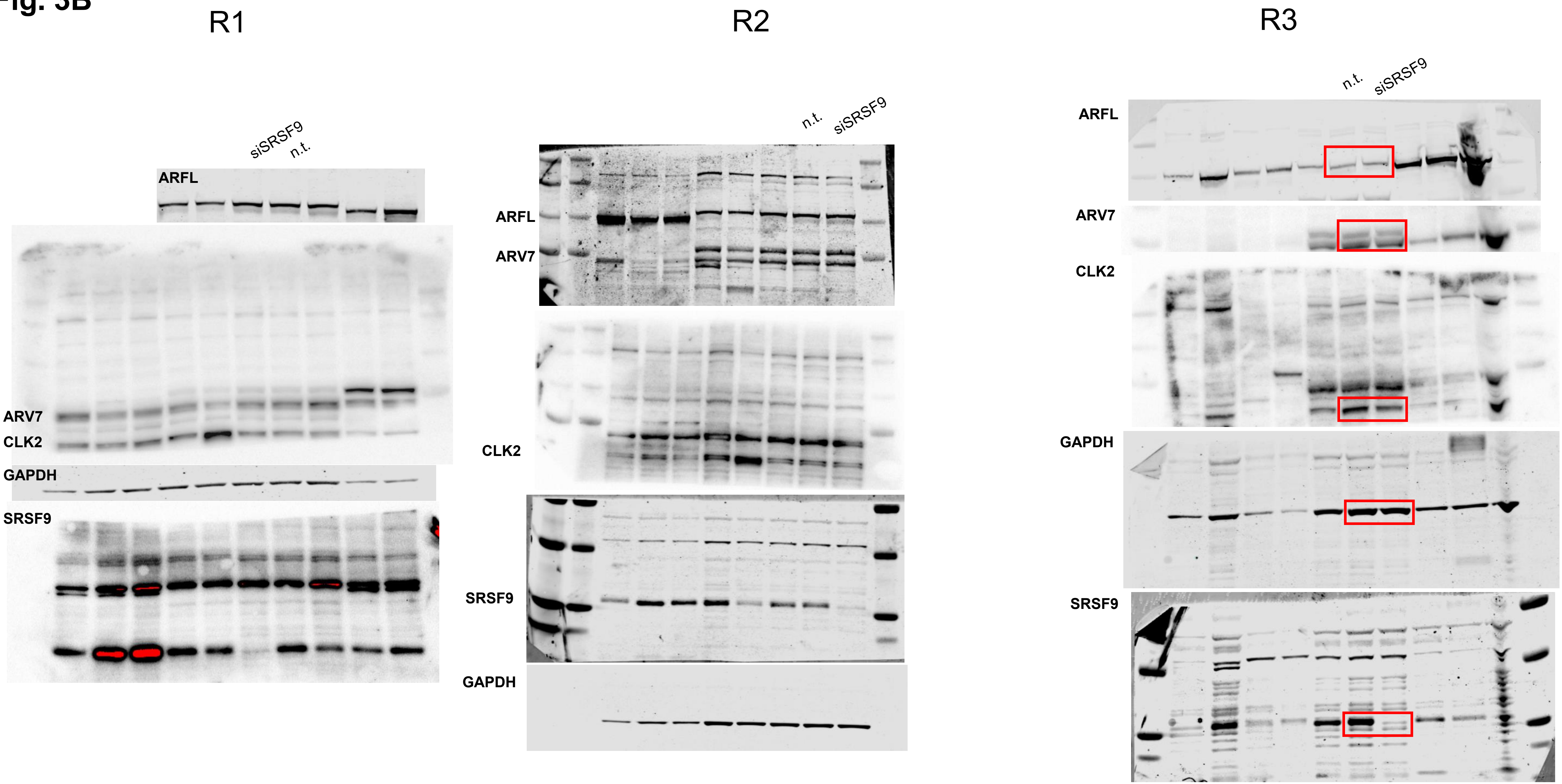

R1
siSRSF9
n.t.
ARFL
ARV7
CLK2
GAPDH
SRSF9
R2
n.t.
siSRSF9
ARFL
ARV7
CLK2
SRSF9
GAPDH
R3
n.t.
siSRSF9
ARFL
ARV7
CLK2
GAPDH
SRSF9

Fig. 5C

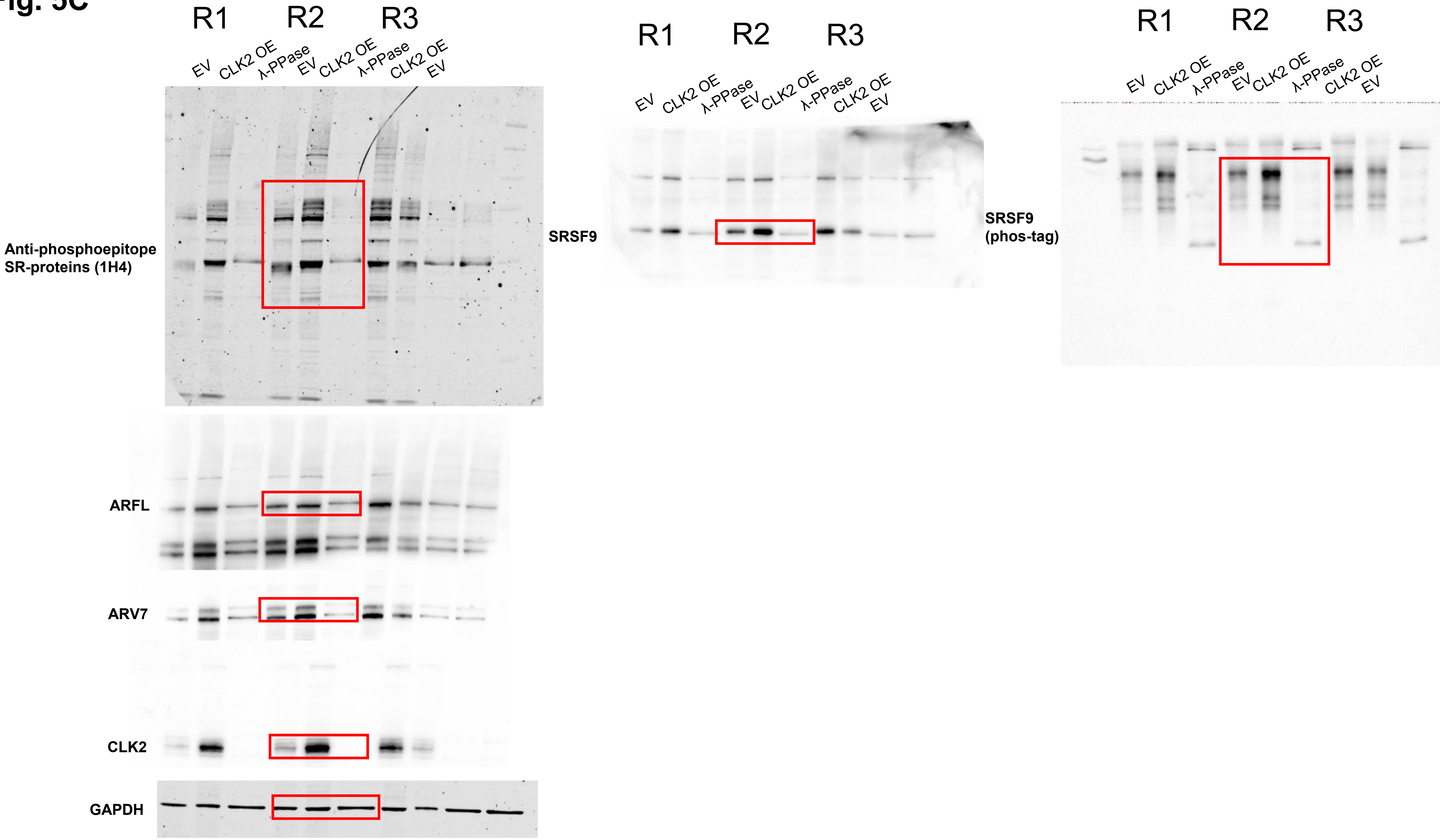
R1
R2
R3
EV
CLK2 OE
λ-PPase
EV
CLK2 OE
λ-PPase
CLK2 OE
EV
Anti-phosphoepitope SR-proteins (1H4)
SRSF9
SRSF9 (phos-tag)
ARFL
ARV7
CLK2
GAPDH

Fig. 6I

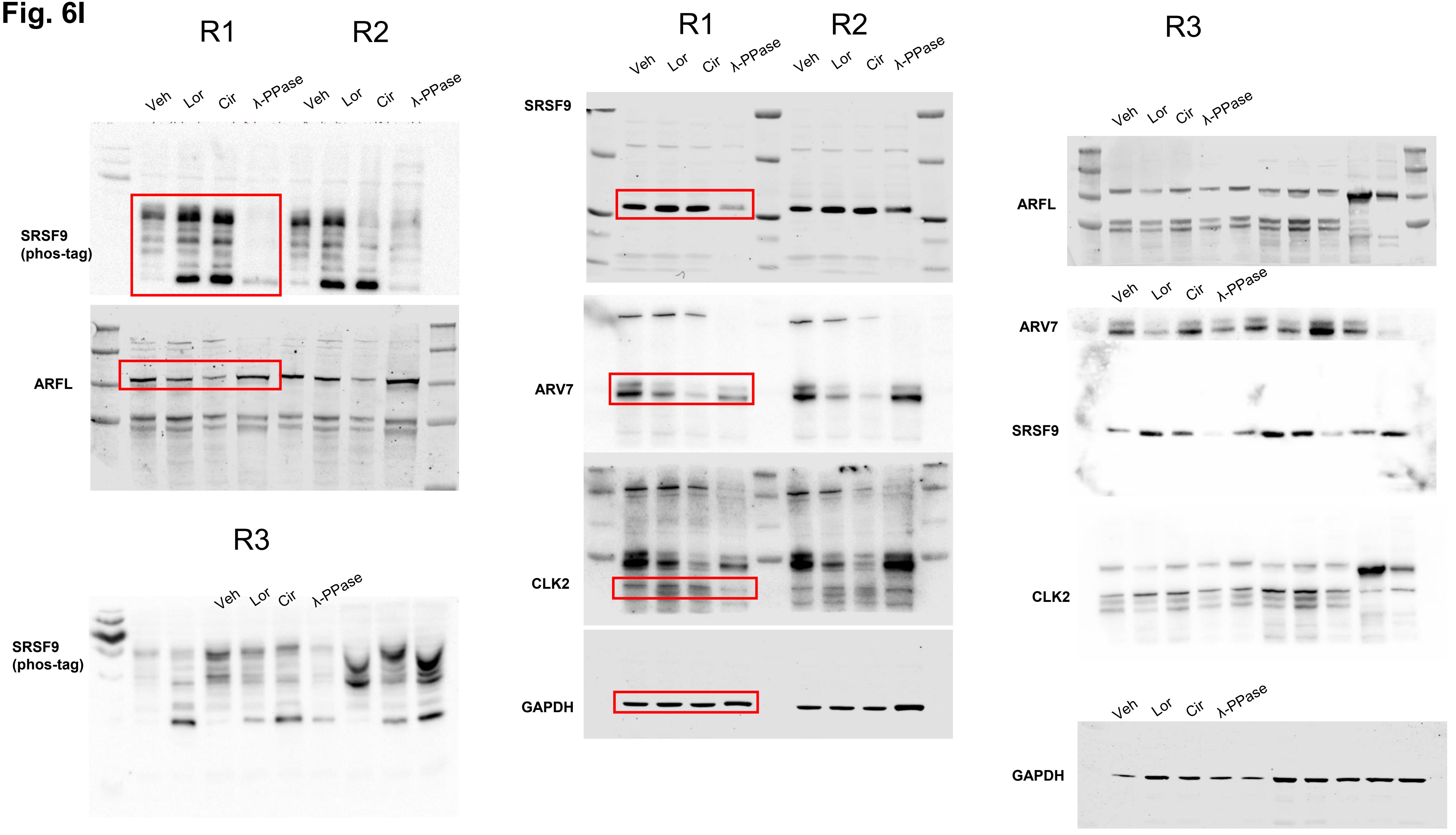
R1
R2
Veh
Lor
Cir
λ-PPase
SRSF9 (phos-tag)
ARFL
R3
SRSF9
ARV7
CLK2
GAPDH

Fig. 6J

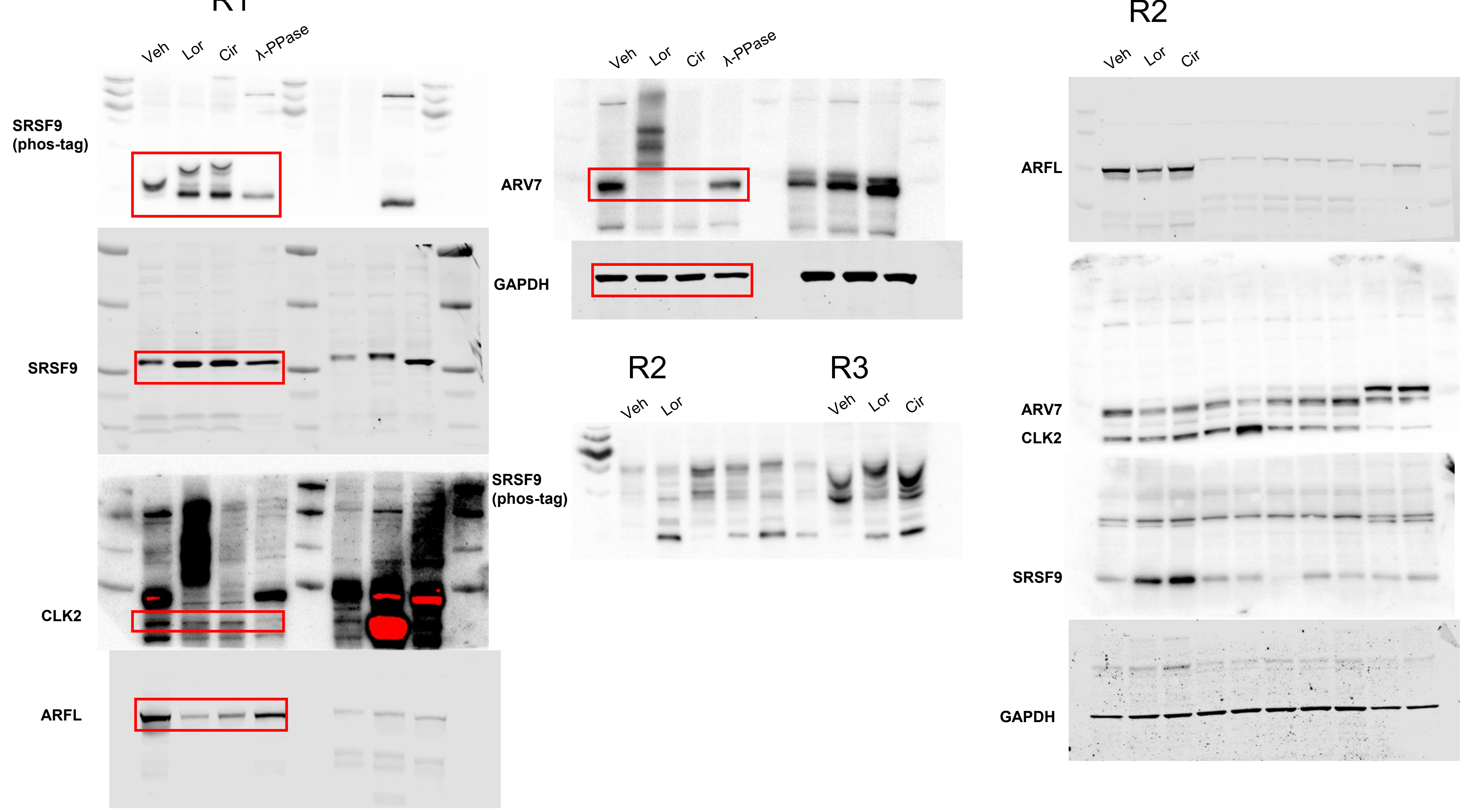
R1
Veh
Lor
Cir
λ-PPase
SRSF9
(phos-tag)
SRSF9
CLK2
ARFL
ARV7
GAPDH
SRSF9
(phos-tag)
R2
R3
ARFL
ARV7
CLK2
SRSF9
GAPDH

Fig. 6J

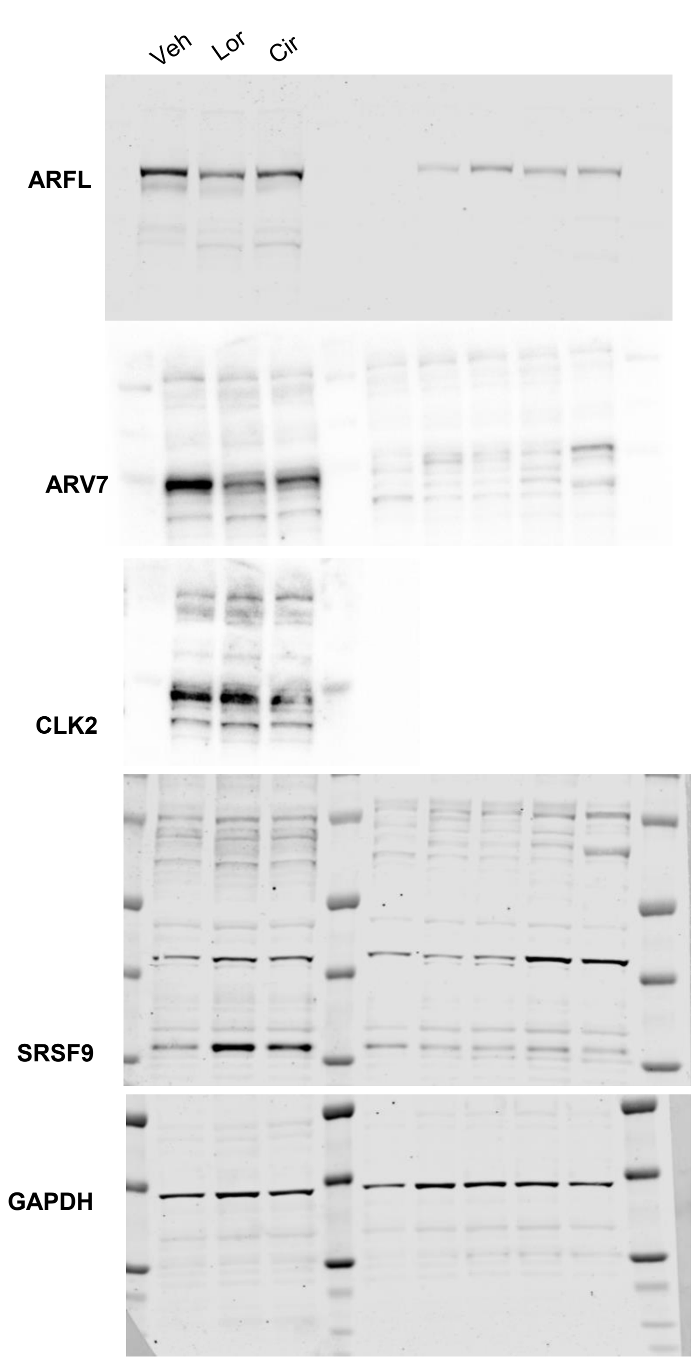
R3
Veh
Lor
Cir
ARFL
ARV7
CLK2
SRSF9
GAPDH

Fig. 7C

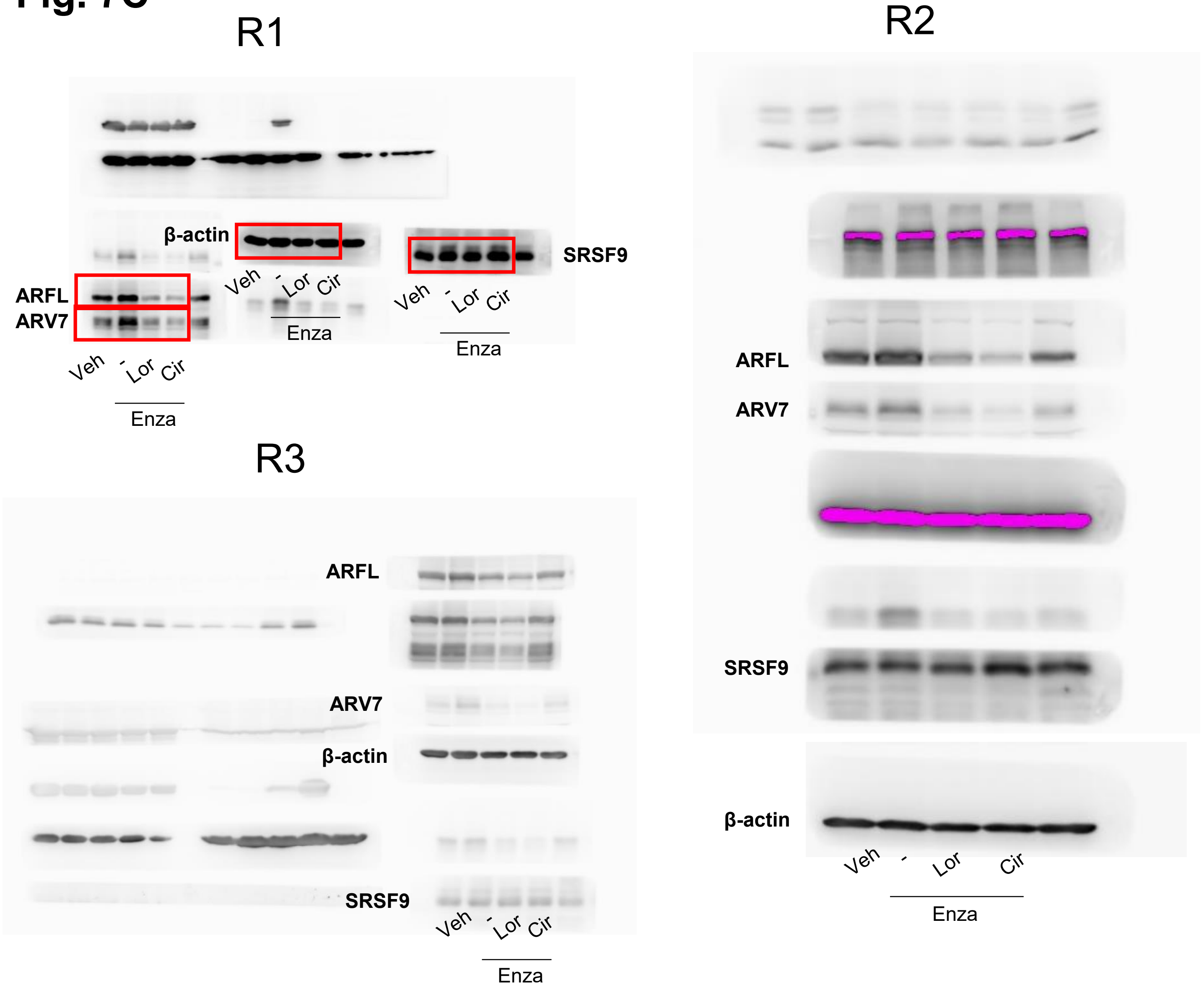
R1
β-actin
SRSF9
ARFL
ARV7
Veh
-
Lor
Cir
Enza
R2
ARFL
ARV7
SRSF9
β-actin
Veh
-
Lor
Cir
Enza
R3
ARFL
ARV7
β-actin
SRSF9
Veh
-
Lor
Cir
Enza
