# Supplementary material for: Targeting the CLK2/SRSF9 splicing axis in prostate cancer leads to decreased ARV7 expression
Source: Mol Oncol. 2024 Sep 11;19(2):496–518. doi: 10.1002/1878-0261.13728 (PMC11792998; doi:10.1002/1878-0261.13728)
Supplement: Supplementary file 1 — Fig. S1. Uncropped immunoblots of this study. Fig. S2. Supporting data for Fig. 1: An intact 3′UTR sequence is important for ARV7 mRNA expression. Fig. S3. Supporting data for Fig. 2: rs5918762 is a common SNP located in the 3′UTR of ARV7 having a role in AR alternative splicing. Fig. S4. Supporting data for Fig. 3: SRSF9 binds to ARV7's 3′UTR in the alternative rs5918762 C allele promoting CE3 inclusion. Fig. S5. Supporting data for Fig. 5: The AR regulates its own splicing by interfering with the CLK2/SRSF9 axis. Fig. S6. Supporting data for Fig. 6: Treatment with the splicing inhibitors Lor and Cir leads to decreased ARV7 expression. Fig. S7. Supporting data for Fig. 7: CLK2 inhibition results in sensitization to Enzalutamide. [file MOL2-19-496-s004.zip › Supplementary_Legends.docx]

***Legends to Supplementary Figures 1-7 and Supplementary Tables 1-6***

**Supplementary Figure 1.** Uncropped, and additional immunoblots underlying the statistical analyses (densitometric quantifications) in this study.

**Supplementary Figure 2:** A) Detailed analysis of the consequences of Cas9/NHEJ-targeting of the 3´UTR of ARV7 in DuCaP. Copy numbers of AR located on chromosome X were assessed in gDNA extracted from DuCaP-cas9 using qPCR. Normalization was done using GAPDH as a reference. gDNA isolated from female or male blood was used as control. DMD, another gene located on chromosome X was determined to differentiate between AR locus or chromosome X amplifications. Copy numbers were assessed after transfection of gV7_4 or non-targeting gRNA. B) Scheme depicting the positions of gRNAs gFL_1 and gFL_2 targeting the 3´UTR of ARFL. C) Dot plots of densitometric quantification of Fig. 1E for ARV7 and ARFL normalized to GAPDH (mean ±SEM, n=3, one-way ANOVA statistical testing). D) Dot plot depicting editing efficiencies determined through Sanger sequencing deconvolution by TIDE analysis in 22Rv1. Examples of two deconvolutions are given. gRNA gV7_1 mediated editing is  pre-dominantly characterized by short deletions, whereas gV7_4 mediated editing is characterized by the insertion of a single nucleotide, explaining the differences in editing efficiency and observed effect on ARV7 expression. E) Dot plots of densitometric  quantification of Fig. 1G for ARV7 and ARFL normalized to GAPDH (mean ±SEM, n=3, one-way ANOVA statistical testing). F) Dot plot depicting editing efficiencies determined through sanger sequencing deconvolution by TIDE analysis in DuCaP. G) Detailed analysis of the consequences of Cas9/NHEJ-targeting of the 3´UTR of ARV7 in 22Rv1. PCR was performed on 22Rv1-cas9 cDNA samples after transfection with non-targeting gRNA or gV7_4. The use of primers on the E2/3 boundary and within CE3 to detect ARV7 yielded in two amplicons corresponding to transcripts containing two copies or a single copy of E3, as identified by Sanger sequencing, respectively. When using primers to detect ARFL (E2/3 and E4/5 boundaries) in the same samples, a single amplicon was observable with the non-targeting gRNA, while, in the case of gV7_4, three distinct bands corresponding to two, one, or none copies of E3 were identifiable. This led us to conclude that gene editing by gV7_4 is able to de-duplicate or delete the 35kb tandem duplication locus (and thus E3) explaining the observed band pattern in ARFL immunoblots (Fig. 1E).

**Supplementary Figure 3:** A) *In silico* predicted RNA secondary structure of the region around rs5918762 in its reference T (left) or alternative C (right) allele using the RNAfold plugin (ViennaRNA package 2.0) of Snapgene. Inlays depict the predicted RNA folding of the whole 3´UTR of *ARV7* mRNA. B) Dual Luciferase Glo assay using ARV7’s UTR cloned 3´ to the Firefly coding sequence. Firefly activity normalized to Renilla activity was assessed for different SNPs in their alternative allele compared to their reference allele in HEK293FT cells. (mean ±SEM, n=4, one-way ANOVA statistical testing*).*

**Supplementary Figure 4.** Dot plots of densitometric quantification of Fig. 3B for ARV7, ARFL, and SRSF9 normalized to GAPDH (mean ±SEM, n=3, Student´s t-test statistical testing).

**Supplementary Figure 5.** A) Dot plots of densitometric quantification of Fig. 5C for ARV7, ARFL, CLK2, SRSF9, and phosphorylation of SRSF4, SRSF11, and SRSF6 normalized to GAPDH in 22Rv1 (mean ±SEM, n=3, Student's t-test and one-way ANOVA statistical testing). B) qPCR for SRSF9 after stable CLK2 overexpression in 22Rv1 (mean ±SEM, n=3, Student's t-test statistical testing). C) qPCR and immunoblot of CLK2 and SRSF9 after SRSF9 downregulation by RNAi. qPCR and immunoblot analyses were done after transfection with non-targeting siRNA or a pool of 4 siRNAs specific for SRSF9. Dot plot indicates densitometric quantification of CLK2 normalized to GAPDH (mean ±SEM, n=3, Student's t-test statistical testing).

**Supplementary Figure 6.** A) Scheme depicting the generation of MDA PCa 2b Enza-R. B) Volcano plot of differential expressed genes of MDA PCa 2b-EnzaR vs. MDA PCa 2b (parental). C) Sanger sequencing of the rs5918762 locus in gDNA extracted from MDA PCa 2b and LNCaP. ABI traces were aligned to the reference genome using Snapgene. D) Microscope images of parental MDA PCa 2b (vehicle) and MDA PCa 2b-EnzaR cells at 100x and 200x fold magnification. E) Dot plots of densitometric quantification of Fig. 6I-J for ARV7, ARFL, CLK2, and SRSF9 after CLK inhibition by 72 h treatment with Lor or Cir at IC50 concentrations in 22Rv1 (left) and DuCaP (right).  (mean ±SEM, n=3, two-way ANOVA statistical testing). F) Volcano plot of differential expressed genes between Lor (50 nM)-treated and untreated (DMSO) 22Rv1 from the short-read RNA sequencing dataset. G) (left) Gene set enrichment analysis using the Hallmark pathways arranged from the long-read, isoform-specific RNA sequencing dataset of Lor (50 nM)-treated vs. untreated (DMSO) 22Rv1 cells. (right) Heatmap of differential expressed genes of the Hallmark pathway “Androgen response”. Dashed lines indicate significance thresholds of p=0.05.

**Supplementary Figure 7.**  Dot plots of densitometric quantification of Fig. 7B for ARV7, ARFL, and SRSF9 in 22Rv1 (mean ±SEM, n=3, two-way ANOVA statistical testing).

**Supplementary Table 1.** Detailed characteristics of the patient cohort samples used in Fig. 2D & 4C.

**Supplementary Table 2.** Sheet 1: List of differentially expressed genes in MDA PCa 2b- EnzaR vs. parental MDA PCa 2b. Sheet 2: List of gene set enrichment analysis using the Hallmarks pathways.

**Supplementary Table 3.** Sheet 1: List of differentially expressed genes in 22Rv1 cells treated with 50 nM Lor vs. vehicle (DMSO) from short-read RNA sequencing. Sheet 2: List of gene set enrichment analysis using the Hallmarks pathways.

**Supplementary Table 4.** Sheet 1: List of differentially expressed genes in 22Rv1 cells treated with 50 nM Lor vs. vehicle (DMSO) from long-read RNA sequencing. Sheet 2: List of gene set enrichment analysis using the Hallmarks pathways.

**Supplementary Table 5.** List of materials used in this study. Suppliers, product numbers and research identification ID (RRID) are indicated, if available.

**Supplementary Table 6.** Raw data file of all plots in this study.
