# Supplementary figures and images for: Targeting the CLK2/SRSF9 splicing axis in prostate cancer leads to decreased ARV7 expression
Source: Mol Oncol. 2024 Sep 11;19(2):496–518. doi: 10.1002/1878-0261.13728 (PMC11792998; doi:10.1002/1878-0261.13728)

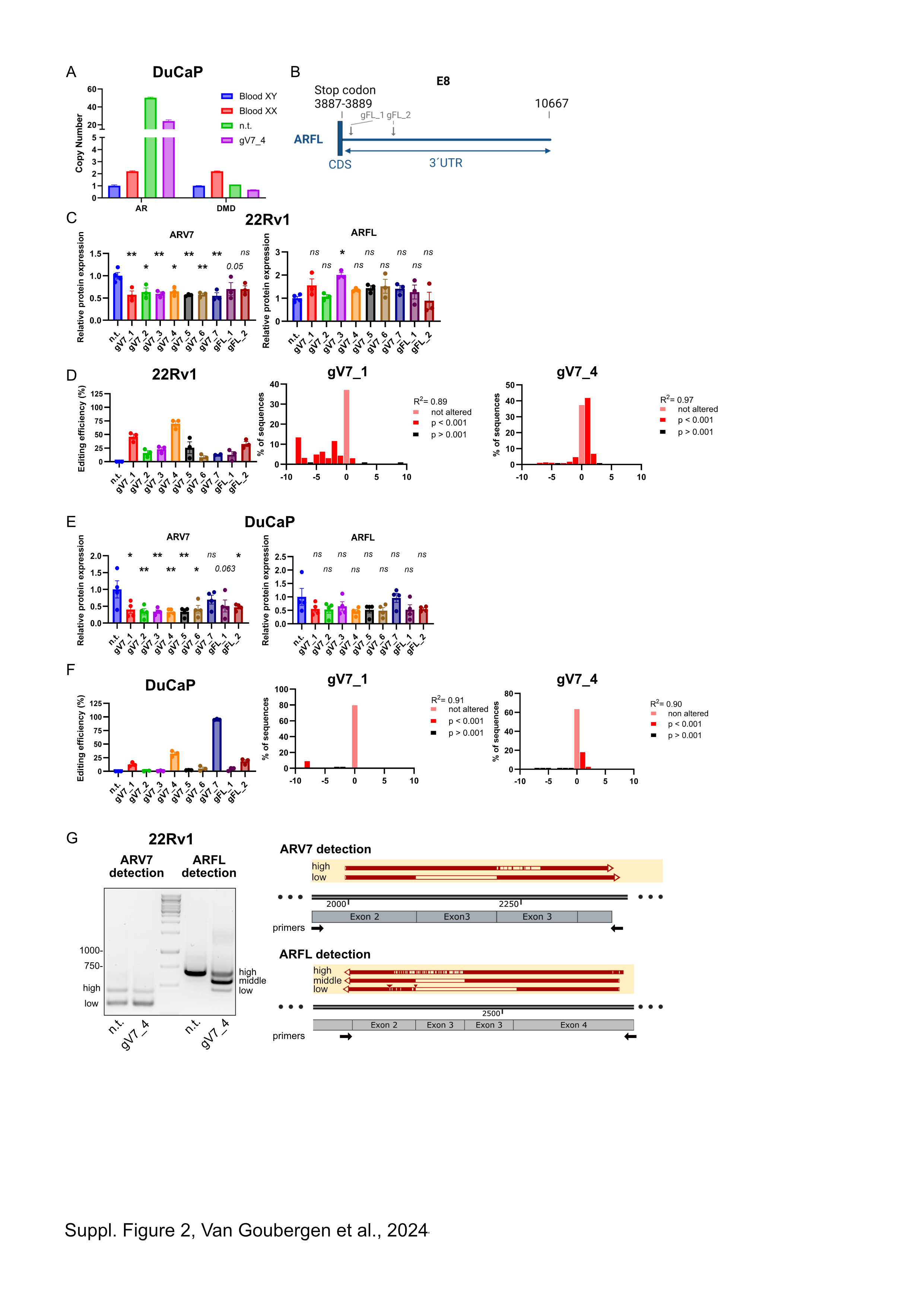

Supplement: Supplementary file 1 — Fig. S1. Uncropped immunoblots of this study. Fig. S2. Supporting data for Fig. 1: An intact 3′UTR sequence is important for ARV7 mRNA expression. Fig. S3. Supporting data for Fig. 2: rs5918762 is a common SNP located in the 3′UTR of ARV7 having a role in AR alternative splicing. Fig. S4. Supporting data for Fig. 3: SRSF9 binds to ARV7's 3′UTR in the alternative rs5918762 C allele promoting CE3 inclusion. Fig. S5. Supporting data for Fig. 5: The AR regulates its own splicing by interfering with the CLK2/SRSF9 axis. Fig. S6. Supporting data for Fig. 6: Treatment with the splicing inhibitors Lor and Cir leads to decreased ARV7 expression. Fig. S7. Supporting data for Fig. 7: CLK2 inhibition results in sensitization to Enzalutamide. [file MOL2-19-496-s004.zip › mol213728-sup-0002-FigS2.tiff]

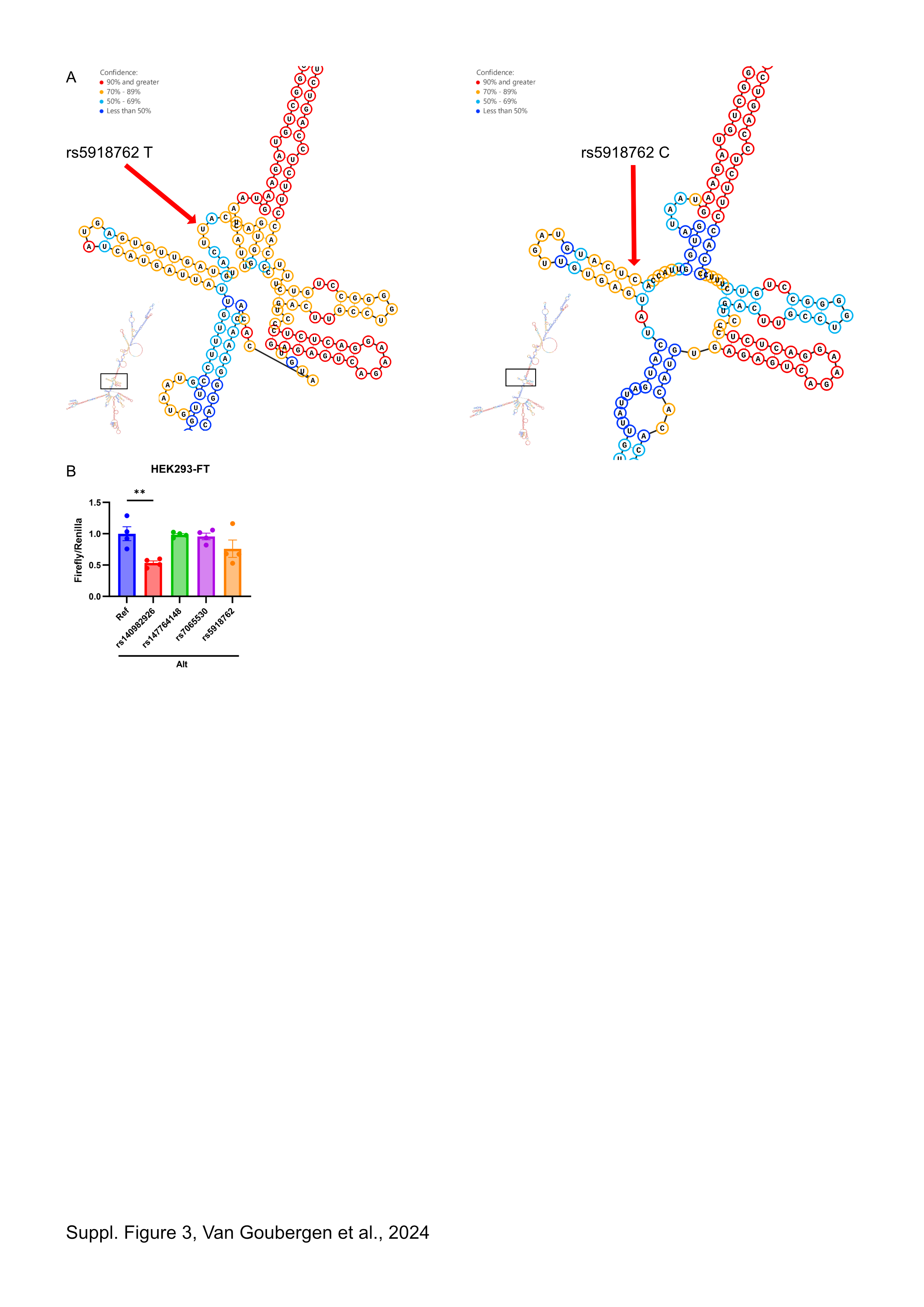

Supplement: Supplementary file 1 — Fig. S1. Uncropped immunoblots of this study. Fig. S2. Supporting data for Fig. 1: An intact 3′UTR sequence is important for ARV7 mRNA expression. Fig. S3. Supporting data for Fig. 2: rs5918762 is a common SNP located in the 3′UTR of ARV7 having a role in AR alternative splicing. Fig. S4. Supporting data for Fig. 3: SRSF9 binds to ARV7's 3′UTR in the alternative rs5918762 C allele promoting CE3 inclusion. Fig. S5. Supporting data for Fig. 5: The AR regulates its own splicing by interfering with the CLK2/SRSF9 axis. Fig. S6. Supporting data for Fig. 6: Treatment with the splicing inhibitors Lor and Cir leads to decreased ARV7 expression. Fig. S7. Supporting data for Fig. 7: CLK2 inhibition results in sensitization to Enzalutamide. [file MOL2-19-496-s004.zip › mol213728-sup-0003-FigS3.tiff]

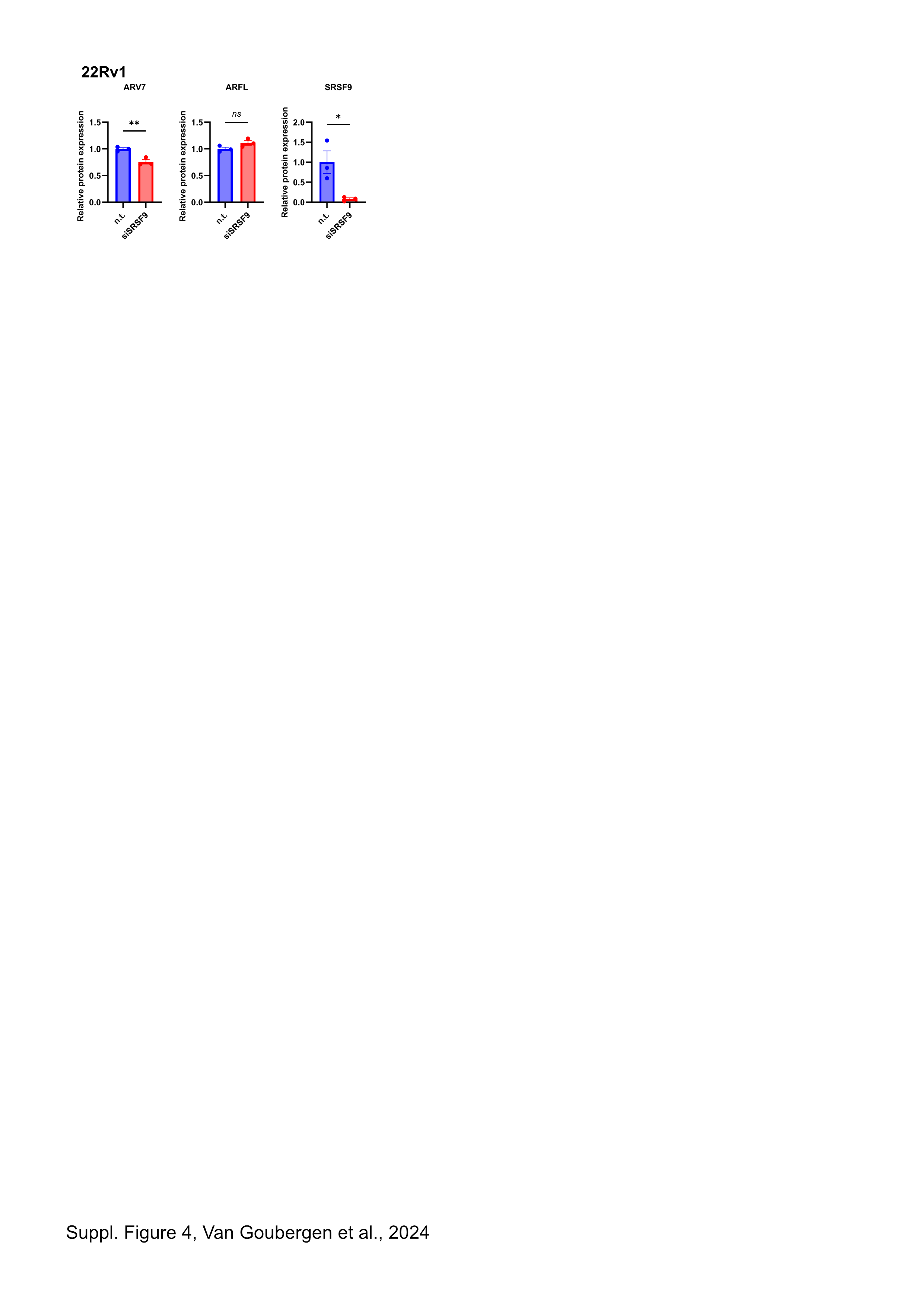

Supplement: Supplementary file 1 — Fig. S1. Uncropped immunoblots of this study. Fig. S2. Supporting data for Fig. 1: An intact 3′UTR sequence is important for ARV7 mRNA expression. Fig. S3. Supporting data for Fig. 2: rs5918762 is a common SNP located in the 3′UTR of ARV7 having a role in AR alternative splicing. Fig. S4. Supporting data for Fig. 3: SRSF9 binds to ARV7's 3′UTR in the alternative rs5918762 C allele promoting CE3 inclusion. Fig. S5. Supporting data for Fig. 5: The AR regulates its own splicing by interfering with the CLK2/SRSF9 axis. Fig. S6. Supporting data for Fig. 6: Treatment with the splicing inhibitors Lor and Cir leads to decreased ARV7 expression. Fig. S7. Supporting data for Fig. 7: CLK2 inhibition results in sensitization to Enzalutamide. [file MOL2-19-496-s004.zip › mol213728-sup-0004-FigS4.tiff]

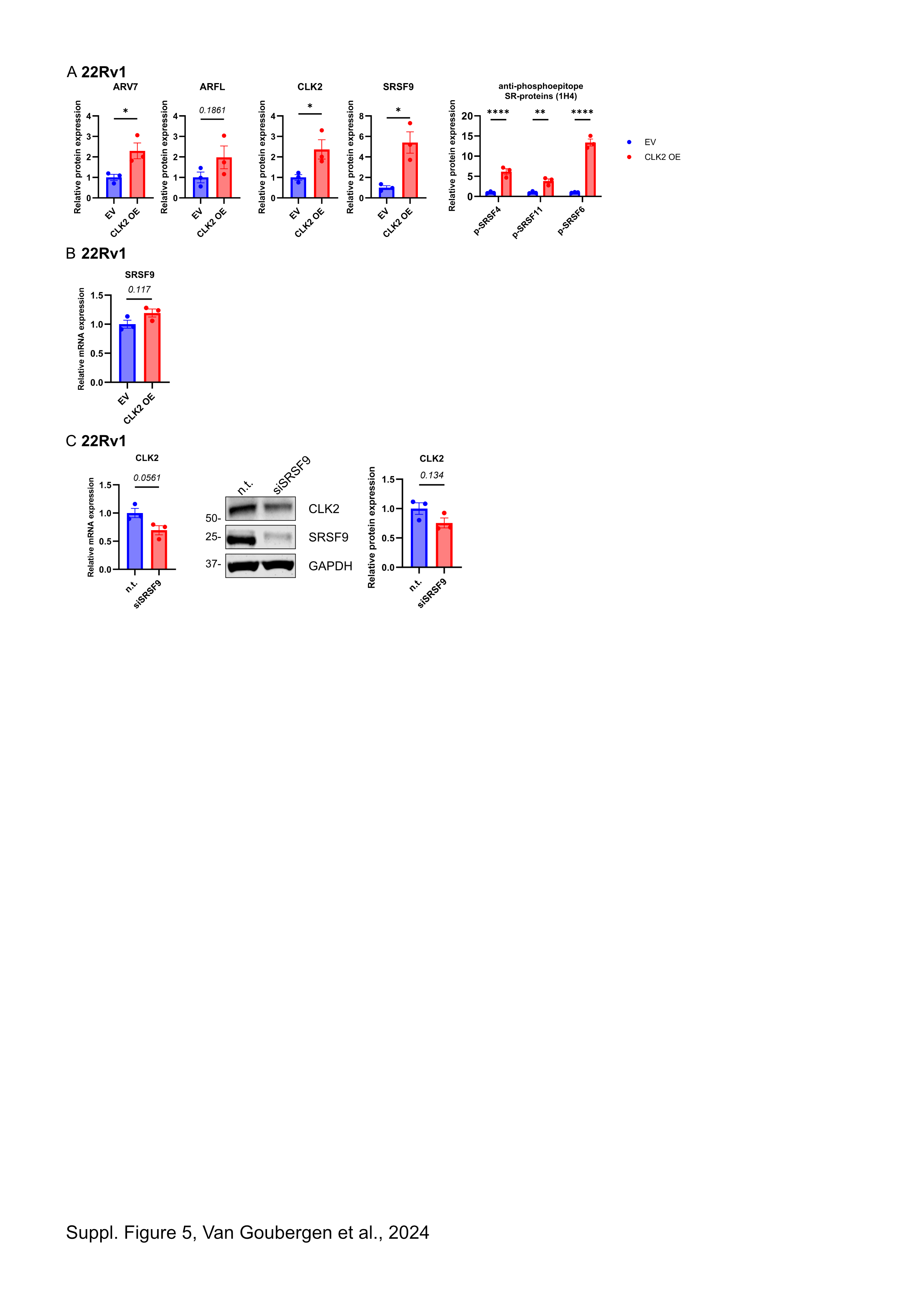

Supplement: Supplementary file 1 — Fig. S1. Uncropped immunoblots of this study. Fig. S2. Supporting data for Fig. 1: An intact 3′UTR sequence is important for ARV7 mRNA expression. Fig. S3. Supporting data for Fig. 2: rs5918762 is a common SNP located in the 3′UTR of ARV7 having a role in AR alternative splicing. Fig. S4. Supporting data for Fig. 3: SRSF9 binds to ARV7's 3′UTR in the alternative rs5918762 C allele promoting CE3 inclusion. Fig. S5. Supporting data for Fig. 5: The AR regulates its own splicing by interfering with the CLK2/SRSF9 axis. Fig. S6. Supporting data for Fig. 6: Treatment with the splicing inhibitors Lor and Cir leads to decreased ARV7 expression. Fig. S7. Supporting data for Fig. 7: CLK2 inhibition results in sensitization to Enzalutamide. [file MOL2-19-496-s004.zip › mol213728-sup-0005-FigS5.tiff]

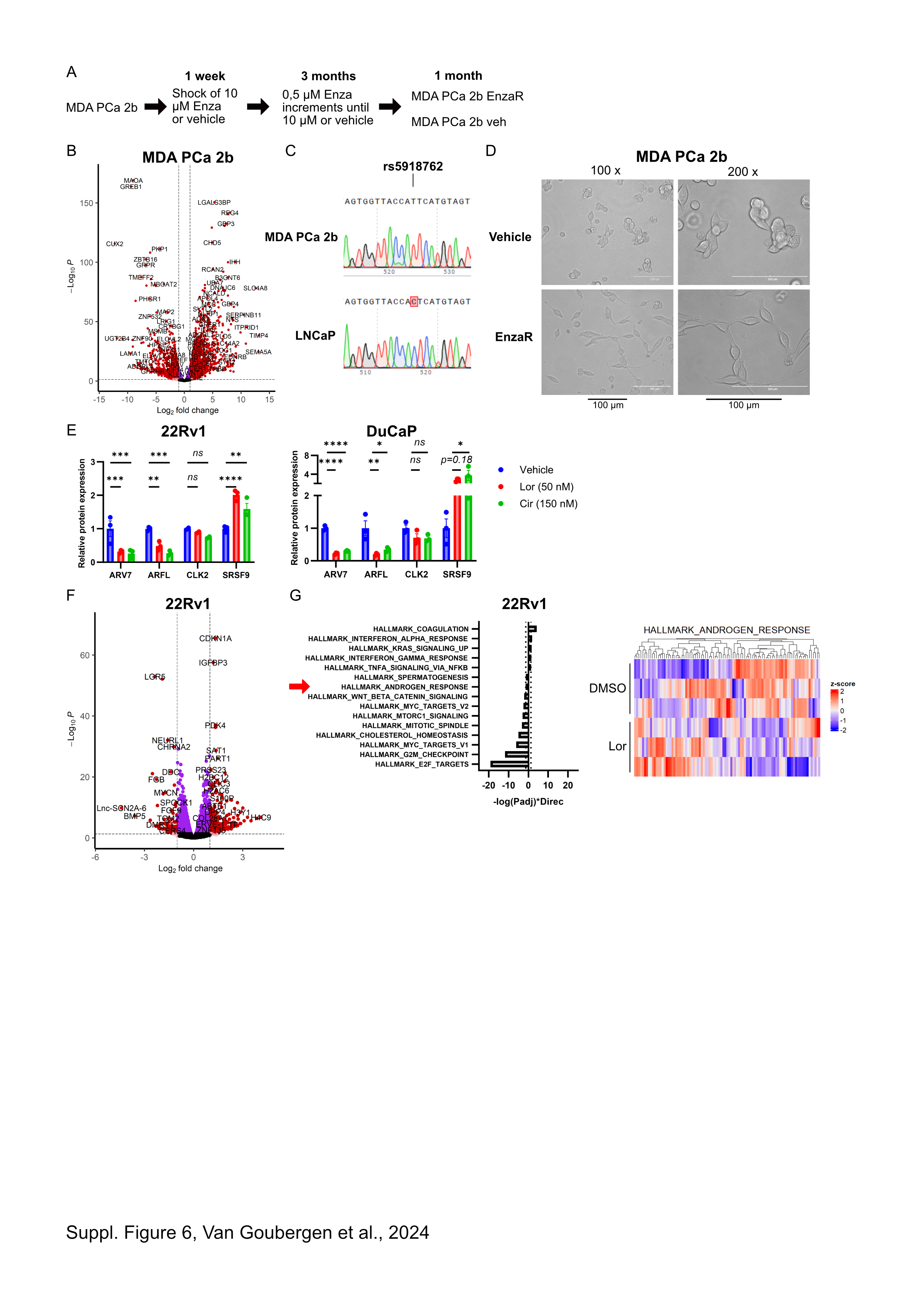

Supplement: Supplementary file 1 — Fig. S1. Uncropped immunoblots of this study. Fig. S2. Supporting data for Fig. 1: An intact 3′UTR sequence is important for ARV7 mRNA expression. Fig. S3. Supporting data for Fig. 2: rs5918762 is a common SNP located in the 3′UTR of ARV7 having a role in AR alternative splicing. Fig. S4. Supporting data for Fig. 3: SRSF9 binds to ARV7's 3′UTR in the alternative rs5918762 C allele promoting CE3 inclusion. Fig. S5. Supporting data for Fig. 5: The AR regulates its own splicing by interfering with the CLK2/SRSF9 axis. Fig. S6. Supporting data for Fig. 6: Treatment with the splicing inhibitors Lor and Cir leads to decreased ARV7 expression. Fig. S7. Supporting data for Fig. 7: CLK2 inhibition results in sensitization to Enzalutamide. [file MOL2-19-496-s004.zip › mol213728-sup-0006-FigS6.tiff]

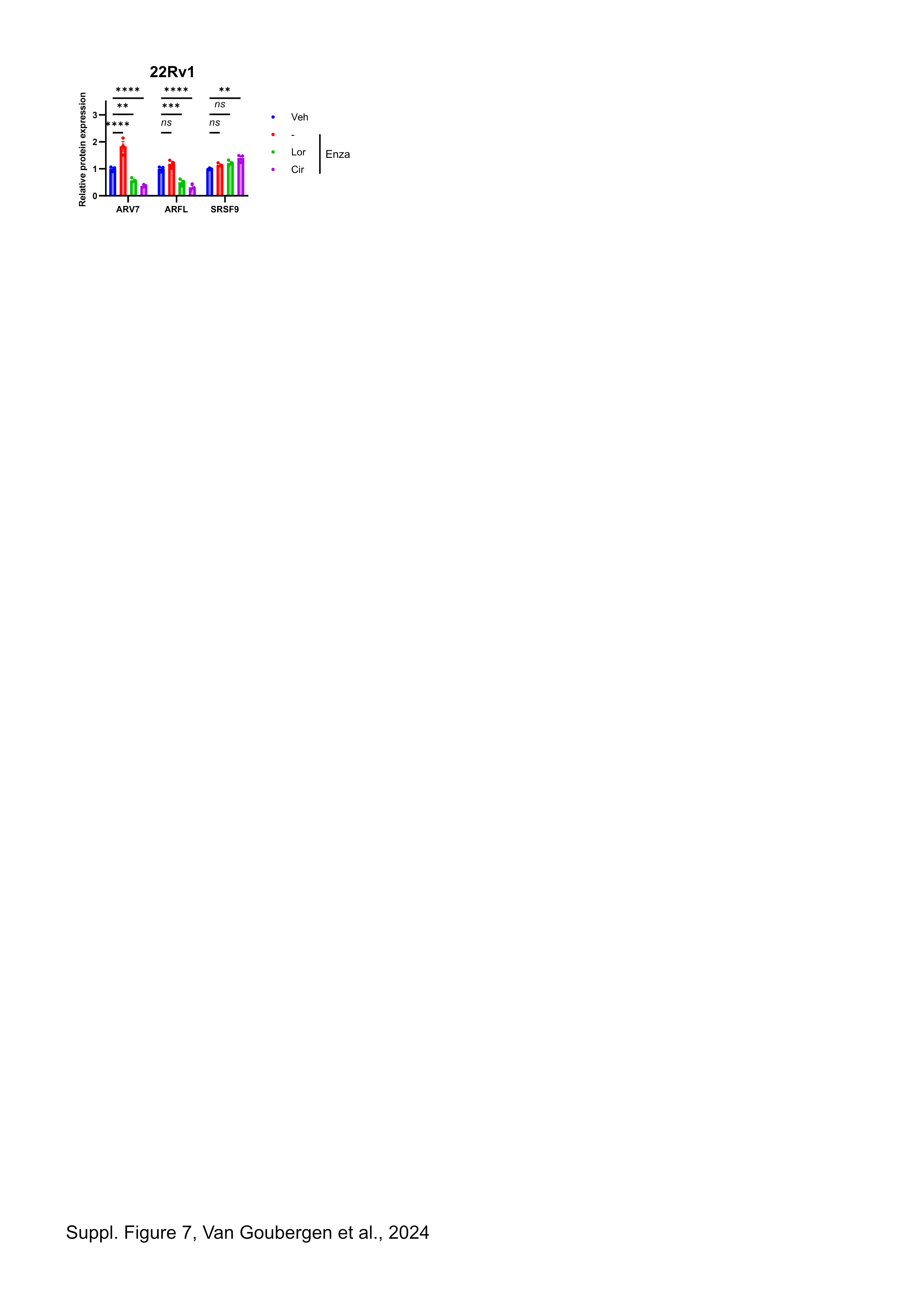

Supplement: Supplementary file 1 — Fig. S1. Uncropped immunoblots of this study. Fig. S2. Supporting data for Fig. 1: An intact 3′UTR sequence is important for ARV7 mRNA expression. Fig. S3. Supporting data for Fig. 2: rs5918762 is a common SNP located in the 3′UTR of ARV7 having a role in AR alternative splicing. Fig. S4. Supporting data for Fig. 3: SRSF9 binds to ARV7's 3′UTR in the alternative rs5918762 C allele promoting CE3 inclusion. Fig. S5. Supporting data for Fig. 5: The AR regulates its own splicing by interfering with the CLK2/SRSF9 axis. Fig. S6. Supporting data for Fig. 6: Treatment with the splicing inhibitors Lor and Cir leads to decreased ARV7 expression. Fig. S7. Supporting data for Fig. 7: CLK2 inhibition results in sensitization to Enzalutamide. [file MOL2-19-496-s004.zip › mol213728-sup-0007-FigS7.tiff]
